# Bayesian estimation of the discrete coefficient of determination

**DOI:** 10.1186/s13637-015-0035-4

**Published:** 2016-01-15

**Authors:** Ting Chen, Ulisses M. Braga-Neto

**Affiliations:** 1 Emmes Corporation, 401 N. Washington Street, Suite 700, Rockville, 20850 MD USA; 2 Department of Electrical and Computer Engineering, Texas A&M University, College Station, 77843 TX, USA

**Keywords:** Discrete coefficient of determination, Bayesian inference, Gene regulatory network inference

## Abstract

The discrete coefficient of determination (CoD) measures the nonlinear interaction between discrete predictor and target variables and has had far-reaching applications in Genomic Signal Processing. Previous work has addressed the inference of the discrete CoD using classical parametric and nonparametric approaches. In this paper, we introduce a Bayesian framework for the inference of the discrete CoD. We derive analytically the optimal minimum mean-square error (MMSE) CoD estimator, as well as a CoD estimator based on the Optimal Bayesian Predictor (OBP). For the latter estimator, exact expressions for its bias, variance, and root-mean-square (RMS) are given. The accuracy of both Bayesian CoD estimators with non-informative and informative priors, under fixed or random parameters, is studied via analytical and numerical approaches. We also demonstrate the application of the proposed Bayesian approach in the inference of gene regulatory networks, using gene-expression data from a previously published study on metastatic melanoma.

## Introduction

DNA regulatory circuits can be often described by networks of Boolean logical gates updated and observed at discrete time intervals [1–6]. In a stochastic setting, the degree of association between Boolean predictors and targets can be quantified by means of the discrete coefficient of determination (CoD) [7]. As such, the CoD is a function of the joint probability of target and predictor variables, which, however, is usually unknown in practice. Hence, this requires the inference of the discrete CoD- given sample data. A larger sample-based CoD value indicates a tighter regulation between target and predictors.

The concept of CoD has far-reaching applications in genomics. The CoD was perhaps the first predictive paradigm utilized in the context of microarray data, the goal being to provide a measure of nonlinear interaction among genes [7]. The CoD has been used in the reconstruction or inference of gene regulatory networks using gene expression data quantized into discrete levels [8–11]. It has also been used in the definition of the intrinsically-multivariate prediction (IMP) criterion for the characterization of canalizing genes [12, 13]. In [14–16], we studied the inferential theory of the discrete CoD in a classical framework, by means of nonparametric and parametric maximum-likelihood estimation (MLE) approaches.

Classical parametric and nonparametric approaches to CoD estimation have been investigated in [14, 15]. In the present paper, we introduce a fully Bayesian approach to the inference of the discrete CoD, based on a parameterized family of target-predictor distributions. Given the priors, the probability model and sample data, we obtain the posterior distributions of the parameters, which can then be used to obtain the optimal predictors and prediction error estimators for the given problem. Such a Bayesian approach for prediction error estimation was first introduced in [17, 18], in a classification context.

Part of the work presented here appeared in [19], which introduced the *minimum mean-square error (MMSE) Bayesian CoD estimator*. In the present paper, we provide an exact representation of the analytical expressions of this estimator, and in addition, introduce the *optimal Bayesian predictor (OBP) CoD estimator*, which is based on an optimal predictor with the minimum expected true error with respect to the posterior distributions of the parameters [20, 21]. We derive exact formulas for the bias, variance, and root-mean-square (RMS) error of the OBP CoD estimator. The accuracy of both Bayesian CoD estimators is compared against that of several nonparametric CoD estimators by numerical simulations. The results indicate that the Bayesian MMSE CoD estimator is the best one when averaged over all distributions and samples, whereas the simpler OBP CoD estimator, though suboptimal in the MMSE sense, can be more accurate than the MMSE CoD estimator, in a frequentist sense, under low-variance informative priors around fixed parameters corresponding to a fixed distribution between target and predictors. It is also unsurprisingly found that priors with higher densities around true fixed distributions produce more accurate Bayesian estimators in a frequentist sense.

This paper is organized as follows. In Section 2, we introduce the discrete model for prediction and present the coefficient of determination in this model. In Section 3, we develop a Bayesian framework of the inference of the discrete CoD, define two Bayesian CoD estimators, one in the sense of minimum mean-square error (MMSE), and the other based on the optimal Bayesian classifier, and derive the analytical expressions for both Bayesian CoD estimators. In Section 4, we first present an exact formulation of accuracy metrics for the OBP CoD estimator. Afterwards, we discuss the accuracy of both Bayesian CoD estimators when averaged over all distributions and samples as well as under fixed distributions under varying priors, and their comparison with the nonparametric CoD estimators. Section 6 describes an approach to the inference of gene regulatory networks using the proposed Bayesian CoD estimators and illustrates the approach with gene expression data from a previously published study on metastatic melanoma. Finally, Section 7 presents concluding remarks.

## The discrete coefficient of determination

The CoD, which was originally defined in classical regression analysis, gives the relative decrease in unexplained variability when entering a variable *X* into the regression of the dependent variable *Y*, in comparison with the total unexplained variability when entering no variables. Dougherty and collaborators extended the concept of CoD to discrete random variables [7]. Given a specified error criterion, such as the mean-square error or the mean-absolute error, the CoD was defined in [7] as

(1) $$ \text{CoD} \,=\, \frac{\varepsilon_{0}-\varepsilon}{\varepsilon_{0}},   $$

where *ε*_0_ is the minimum error of predicting *Y* by a constant (i.e., in the absence of observations) and *ε* is the minimum error of predicting *Y* based on the observation of *X*. Since *ε*≤*ε*_0_ (all sensible error criteria satisfy this property), the CoD ranges from 0 to 1. The closer it is to one, the closer *ε* is to zero and the tighter the association between predictor and target variables, whereas the closer it is to zero, the closer *ε* is to *ε*_0_ and the weaker the association is. By convention, CoD=0 when *ε*_0_=0. The CoD is a function only of the distribution of (*X,Y*); in particular, it is not a function of sample data. This definition of the CoD reduces gracefully to the classical one in the case when (*X,Y*) is jointly Gaussian [7].

We consider in this paper the case where $\mathbf {X} = (X_{1}, X_{2}, \dots, X_{d}) \in \{0,1\}^{d}$ is a binary vector of predicting variables and *Y*∈{0,1} is a binary target random variable. For example, **X** and *Y* may consist of the active/inactive expression state of various genes. The probability distribution of the pair (**X,Y**) is specified by the probability *c*=*P*(*Y*=0), and the probabilities *p*_*i*_=*P*(**X**=**x** ^*i*^∣*Y*=0) and *q*_*i*_=*P*(**X**=**x** ^*i*^∣*Y*=1), for $ i = 1, \dots, b$, with $\sum _{i=1}^{2^{d}} p_{i} = 1$ and $\sum _{i=1}^{2^{d}} q_{i} = 1$. Let $\left (\mathbf {x}^{1},\ldots,\mathbf {x}^{2^{d}}\right)$ be an arbitrary enumeration of the possible values of the predicting vector **X**. An optimal predictor of *Y* given **X** is well-known to be *ψ* ^∗^(**X**)= arg max*k* *P*(*Y*=*k*∣**X**) [22]. The minimum error of predicting *Y* based on the observation of **X** is therefore

(2) $${} {\fontsize{9.4pt}{9.6pt}{\begin{aligned} \varepsilon &\,=\, P(Y \neq \psi^{*}(\mathbf{X})) \,=\, E\left[\min\{P(Y=1\mid X),P(Y=0\mid X)\}\right]\\ & \,=\, \sum_{i=1}^{2^{d}} \min\left\{P\left(Y=1 \mid \mathbf{X} = \mathbf{x}^{i}\right),P\left(Y=0 \mid \mathbf{X} = \mathbf{x}^{i}\right)\right\}\\&\quad \times P\left(\mathbf{X} = \mathbf{x}^{i}\right) \\ & \,=\, \sum_{i=1}^{2^{d}} \min\left\{P\left(Y=1, \mathbf{X} = \mathbf{x}^{i}\right),P\left(Y=0, \mathbf{X} = \mathbf{x}^{i}\right)\right\} \\ & \,=\, \sum_{i=1}^{2^{d}} \min\left\{P\left(\mathbf{X} = \mathbf{x}^{i}\mid Y=1\right)P(Y=1), P(\mathbf{X} = \mathbf{x}^{i} \mid\right. \\&\qquad\qquad\qquad\left. Y=0)P(Y=0)\vphantom{\left\{P\left(\mathbf{X} = \mathbf{x}^{i}\mid Y=1\right)P(Y=1), P(\mathbf{X} = \mathbf{x}^{i} \mid\right.}\right\} \\ &\,=\, \sum_{i=1}^{2^{d}} \min\left\{c\,p_{i},(1-c)q_{i}\right\}\\& \,=\, \sum_{i=1}^{2^{d}} \left(c\,p_{i} \,I_{p_{i} < \frac{1-c}{c} q_{i}}+ (1-c) q_{i} \,I_{q_{i} \leq \frac{c}{1-c}p_{i}}\right), \end{aligned}}}   $$

where *I*_*A*_ is the indicator function, which is equal to 1 if A is satisfied and zero, otherwise. On the other hand, an optimal predictor in the absence of observations is clearly given by *ψ* ^∗^= arg max*k* *P*(*Y*=*k*), so that the minimum error of predicting *Y* by a constant is given by

(3) $$ \varepsilon_{0} \,=\, \min\{P(Y=0), P(Y=1)\} \,=\, \min\{c,1-c\},   $$

Plugging (2) and (3) in (1) results in

(4) $$ \begin{aligned} &\text{CoD} \,=\, 1-\sum_{i=1}^{2^{d}} \left(\frac{c}{\min\{c,1\,-\,c\}}\, p_{i} \,I_{p_{i} < \frac{1-c}{c} q_{i}} \right.\\&\qquad\qquad\qquad\quad \left. + \frac{1-c}{\min\{c,1\,-\,c\}}\,q_{i} \,I_{q_{i} \leq \frac{c}{1-c}p_{i}}\right),  \end{aligned}  $$

This formula gives the relationship between the CoD and the parameters of the distribution of (**X,Y**).

## Bayesian CoD estimators

In practice, the distributional parameters are generally unknown, and one would like to estimate the CoD from sample data. We present in this section the derivation of two Bayesian estimators for the CoD in (4). One approach is analogous to that followed by [17] in defining the Bayesian MMSE prediction error estimator, whereas the other one makes use of the *optimal Bayesian predictor* (OBP), a straightforward generalization of the optimal Bayesian classifier (OBC), introduced in [20].

We will assume that an i.i.d. sample **S**_*n*_={(**X**_1_,*Y*_1_),…,(**X**_*n*_,*Y*_*n*_)} from the distribution of (**X,Y**) is available. Given **S**_*n*_, define *U*_*i*_ as the number of sample points such that **X**=**x** ^*i*^ and *Y*=0, and *V*_*i*_ as the number of sample points such that **X**=**x** ^*i*^ and *Y*=1, for $i = 1, \dots,2^{d}$. Note that $N_{0} = \sum _{i=1}^{2^{d}} U_{i}$ and $N_{1} = \sum _{i=1}^{2^{d}} V_{i}$ are the (random) sample sizes corresponding to *Y*=0 and *Y*=1, respectively.

Let $\phantom {\dot {i}\!}{\mathbf p} = (p_{1}, \ldots, p_{2^{d}})$, $\phantom {\dot {i}\!}{\mathbf q} = (q_{1},\ldots,q_{2^{d}})$, and ***θ***=(*c*,**p**,**q**), where 0≤*c,p*_*i*_,*q*_*i*_≤1, and $\sum _{i=1}^{2^{d}} p_{i} = \sum _{i=1}^{2^{d}} q_{i} =1$. As shown in the previous section, the distribution of (**X,Y**) is completely specified by the parameter vector ***θ***. The Bayesian approach treats ***θ*** as a random variable, the *prior distribution* of which can take advantage of a priori knowledge about the problem. We will assume that *c*, **p**, and **q** are independent, i.e., *f*(***θ***)=*f*(*c*)*f*(**p**)*f*(**q**). It is shown in [17] that this implies that the *posterior distribution* of ***θ*** also factors *f*(***θ***∣**S**_*n*_)=*f*(*c*∣**S**_*n*_)*f*(**p**∣**S**_*n*_)*f*(**q**∣**S**_*n*_).

In this paper, we will employ the standard choice of priors for discrete distributions, namely, the Beta and Dirichlet distributions (c.f. Appendices A and B):

(5) $$ \begin{aligned} c &\sim \text{Beta}(\alpha,\beta),\\ {\mathbf p} &\sim \text{Dirichlet}(\alpha_{1}, \dots, \alpha_{2^{d}}),\\ {\mathbf q} &\sim \text{Dirichlet}(\beta_{1}, \dots, \beta_{2^{d}}), \end{aligned}   $$

where the hyperparameters *α*, *β*, *α*_*i*_, *β*_*i*_, *i*=1,…,2^*d*^, are positive numbers. These distributions have bounded supports; the Beta distribution is defined over the interval [0,1], while the Dirichlet distribution is defined over the simplex of 2^*d*^ nonnegative numbers that add up to one. The shapes of the distributions are controlled by the *concentration parameters* *Δ*_*c*_=*α*+*β*, $\Delta _{p} = \sum _{j=1}^{2^{d}} \alpha _{j}$, and $\Delta _{q} = \sum _{j=1}^{2^{d}} \beta _{j}$, and the *base measures* *c*_0_=*α*/*Δ*_*c*_, ${\mathbf p}_{0} = (\alpha _{1}/\Delta _{p}, \dots, \alpha _{2^{d}}/\Delta _{p})$, and ${\mathbf q}_{0} = (\beta _{1}/\Delta _{q}, \dots, \beta _{2^{d}}/\Delta _{q})$. Please refer to Appendices A and B for definitions and important facts about the Beta and Dirichlet distributions, which will be needed in the sequel.

A very important property for our purposes is that the Beta and Dirichlet priors are *conjugate priors* for the discrete multinomial distribution, i.e., they have the same form as the corresponding posteriors. Given the sample data **S**_*n*_, the posterior distributions are [17, 18]:

(6) $$ \begin{aligned} c \mid \mathbf{S}_{n} &\sim \text{Beta}(n_{0}+\alpha,n_{1}+\beta), \\ {\mathbf p} \mid \mathbf{S}_{n} &\sim \text{Dirichlet}(u_{1}+\alpha_{1}, \dots, u_{2^{d}}+\alpha_{2^{d}}),\\ {\mathbf q} \mid \mathbf{S}_{n}&\sim \text{Dirichlet}(v_{1}+\beta_{1}, \dots, v_{2^{d}}+\beta_{2^{d}}), \\ \end{aligned}   $$

where *n*_0_ and *n*_1_ are the observed sample sizes corresponding to *Y*=0 and *Y*=1, respectively, while *U*_*i*_ and *V*_*i*_ are the observed sample values of the random variables *U*_*i*_ and *V*_*i*_, respectively.

### Minimum mean-square error CoD estimator

Given a CoD estimator $\widehat {\text {CoD}}$, consider the mean-square error

(7) $$ \text{MSE} \,=\, E_{\boldsymbol{\theta}, \mathbf{S}_{n}}\left[| \widehat{\text{CoD}} - \text{CoD}|^{2}\right],  $$

The minimum MSE solution, as is well known, is given by the expectation of the CoD according to the posterior distribution of the parameters [23]. This defines the *Bayesian MMSE CoD estimator*:

(8) $$ \widehat{\text{CoD}}_{\text{MMSE}}\,=\, E[\!\text{CoD}\mid \mathbf{S}_{n}] \,=\, E_{\boldsymbol{\theta}\mid \mathbf{S}_{n}}[\!\text{CoD}],   $$

where the CoD is given by (4).

It is well-known that the MMSE estimator $\widehat {\text {CoD}}_{\text {MMSE}}$ not only displays the least root mean-square error (RMS) over the distribution of (***θ***,**S**_*n*_), but it is also an unbiased estimator (however, for a specific model with fixed ***θ***, $\widehat {\text {CoD}}_{\text {MMSE}}$ might not be unbiased or have the least RMS).

In order to derive an expression for the Bayesian MMSE CoD estimator, first note that (4) can be rewritten as

(9) $${} {\fontsize{8.6pt}{9.6pt}{\begin{aligned} \text{CoD} &\,=\, 1-\sum_{i=1}^{2^{d}} \left(p_{i} \,I_{p_{i} < \frac{1-c}{c} q_{i}}I_{c<1/2} + \frac{c}{1\,-\,c}\,p_{i} \,I_{p_{i} < \frac{1-c}{c} q_{i}}\,I_{c\geq 1/2}\right. \\ &\quad\quad\quad\quad\quad \left. + \frac{1-c}{c}\,q_{i} \,I_{q_{i} \leq \frac{c}{1-c}p_{i}}I_{c<1/2}+ q_{i} \,I_{q_{i} \leq \frac{c}{1-c}p_{i}}I_{c\geq 1/2}\right)\!. \end{aligned}}}   $$

Applying (8) to (9) and using the previously mentioned fact that the posterior distribution factors allows one to write the Bayesian MMSE CoD estimator as

(10) $${\kern20pt} {\fontsize{9.2pt}{9.6pt}{\begin{aligned} & \widehat{\text{CoD}}_{\text{MMSE}} \,=\, E_{\boldsymbol{\theta}\mid \mathbf{S}_{n}}\left[\text{CoD}\right] \,=\, E_{c\mid\mathbf{S}_{n}}\left[ E_{{\mathbf p}\mid\mathbf{S}_{n}} \left[E_{{\mathbf q}\mid\mathbf{S}_{n}}\left[\text{CoD}\right]\right] \right] \\[-3pt] & \quad =\, 1-\sum_{i=1}^{2^{d}} \left(E_{c\mid\mathbf{S}_{n}} \left[E_{{\mathbf q}\mid\mathbf{S}_{n}}\left[E_{{\mathbf p}\mid\mathbf{S}_{n}}\left[ p_{i} \,I_{p_{i} < \frac{1-c}{c} q_{i}}\right] I_{c<1/2}\right]\right]\right.\\[-3pt] & \quad\quad\quad\quad\quad\:\left. + E_{c\mid\mathbf{S}_{n}} \left[\frac{c}{1\,-\,c}E_{{\mathbf q}\mid\mathbf{S}_{n}} \left[E_{{\mathbf p}\mid\mathbf{S}_{n}} \left[p_{i} \,I_{p_{i} < \frac{1-c}{c} q_{i}}\right] I_{c\geq 1/2}\right]\right]\right. \\[-2pt] & \quad\quad\quad\quad\quad\:\left.+ E_{c\mid\mathbf{S}_{n}} \left[\frac{1\,-\,c}{c}E_{{\mathbf p}\mid\mathbf{S}_{n}} \left[E_{{\mathbf q}\mid\mathbf{S}_{n}} \left[q_{i} \,I_{q_{i} \leq \frac{c}{1-c} p_{i}}\right] I_{c< 1/2}\right]\right]\right. \\[-2pt] & \quad\quad\quad\quad\quad\:\left. +\, E_{c\mid\mathbf{S}_{n}} \left[E_{{\mathbf p}\mid\mathbf{S}_{n}}\left[E_{{\mathbf q}\mid\mathbf{S}_{n}}\left[ q_{i} \,I_{q_{i} \leq \frac{c}{1\,-\,c} p_{i}}\right] I_{c\geq 1/2}\right]\right]\right), \end{aligned}}}   $$

Using (6) and the fact that the marginal distributions of a Dirichlet are Beta (c.f. Appendix B), we have that *c* ∣ **S**_*n*_∼Beta(*α* ^*s*^,*β* ^*s*^), $p_{i} \!\mid \! \mathbf {S}_{n} \sim \text {Beta}({{\alpha ^{s}_{i}}},{{\overline {\alpha }^{\,s}_{i}}})$, and $q_{i} \!\mid \! \mathbf {S}_{n} \sim \text {Beta}({{\beta ^{s}_{i}}},{{\overline {\beta }^{\,s}_{i}}})$, where *α* ^*s*^ = *n*_0_+*α*, *β* ^*s*^=*n*_1_+*β*, ${{\alpha ^{s}_{i}}} = u_{i}+\alpha _{i}$, ${{\overline {\alpha }^{\,s}_{i}}} = n_{0}-u_{i} + \Delta _{p} - \alpha _{i}$, ${{\beta ^{s}_{i}}} = v_{i}+\beta _{i}$, and ${{\overline {\beta }^{\,s}_{i}}} = n_{1}-v_{i} + \Delta _{q} - \beta _{i}$, for *i*=1,…,2^*d*^. Using the results in Appendix A and assuming that the hyperparameters are integers (if they are not, a simple adjustment to the derivation below can be made; see Appendix A), it follows that

(11) $${\kern20pt} {\fontsize{9.2pt}{9.6pt}{\begin{aligned} & E_{c\mid\mathbf{S}_{n}} \left[E_{{\mathbf q}\mid\mathbf{S}_{n}}\left[E_{{\mathbf p}\mid\mathbf{S}_{n}} \left[p_{i} \,I_{p_{i} < \frac{1-c}{c} q_{i}}\right] I_{c<1/2}\right]\right] \,=\, E_{c\mid\mathbf{S}_{n}} \left[E_{{\mathbf q}\mid\mathbf{S}_{n}}\left[E_{{\mathbf p}\mid\mathbf{S}_{n}}\left[p_{i} \,I_{p_{i} < \frac{1-c}{c} q_{i}}\right] I_{q_{i} < \frac{c}{1-c}}\right] I_{c<1/2}\right] \,+\,\\[-2pt] & E_{c\mid\mathbf{S}_{n}} \left[E_{{\mathbf q}\mid\mathbf{S}_{n}}\left[E_{{\mathbf p}\mid\mathbf{S}_{n}}\left[p_{i} \right] \right] I_{c<1/2}\right] \,-\, E_{c\mid\mathbf{S}_{n}} \left[E_{{\mathbf q}\mid\mathbf{S}_{n}}\left[E_{{\mathbf p}\mid\mathbf{S}_{n}}\left[p_{i} \right] I_{q_{i} < \frac{c}{1-c}}\right] I_{c<1/2}\right]\\[-2pt] & =\frac{1}{\mathrm{B}({{\alpha^{s}_{i}}},{{\overline{\alpha}^{\,s}_{i}}})} \times \left\{E_{c\mid\mathbf{S}_{n}} \left[E_{{\mathbf q}\mid\mathbf{S}_{n}}\left[\sum_{j=0}^{{{\overline{\alpha}^{\,s}_{i}}}-1}r_{j}({{\alpha^{s}_{i}}}+1,{{\overline{\alpha}^{\,s}_{i}}}) \left(\frac{1-c}{c}q_{i}\right)^{{{\alpha^{s}_{i}}}+j+1} I_{q_{i} < \frac{c}{1-c}} \right]I_{c<1/2}\right]\,+\, \right.\\[-2pt] & \left.E_{c\mid\mathbf{S}_{n}} \left[\mathrm{B}\left({{\alpha^{s}_{i}}}+1,{{\overline{\alpha}^{\,s}_{i}}}\right) I_{c<1/2} \right] \,-\, E_{c\mid\mathbf{S}_{n}} \left[E_{{\mathbf q}\mid\mathbf{S}_{n}} \left[\mathrm{B}\left({{\alpha^{s}_{i}}}+1,{{\overline{\alpha}^{\,s}_{i}}}\right) I_{q_{i} < \frac{c}{1-c}}\right] I_{c<1/2} \right] \vphantom{\left\{E_{c\mid\mathbf{S}_{n}} \left[E_{{\mathbf q}\mid\mathbf{S}_{n}}\left[\sum_{j=0}^{{{\overline{\alpha}^{\,s}_{i}}}-1}r_{j}({{\alpha^{s}_{i}}}+1,{{\overline{\alpha}^{\,s}_{i}}}) \left(\frac{1-c}{c}q_{i}\right)^{{{\alpha^{s}_{i}}}+j+1} I_{q_{i} < \frac{c}{1-c}} \right]I_{c<1/2}\right]\,+\, \right.}\right\} =\frac{1}{\mathrm{B}\left({{\alpha^{s}_{i}}},{{\overline{\alpha}^{\,s}_{i}}}\right)\mathrm{B}\left({{\beta^{s}_{i}}},{{\overline{\beta}^{\,s}_{i}}}\right)} \times\\ & \left\{ \sum_{j=0}^{{{\overline{\alpha}^{\,s}_{i}}}-1}\:\: \sum_{k=0}^{{{\overline{\beta}^{\,s}_{i}}}-1} r_{j}\left({{\alpha^{s}_{i}}}+1,{{\overline{\alpha}^{\,s}_{i}}}\right)\,r_{k}\left({{\alpha^{s}_{i}}}+{{\beta^{s}_{i}}}+j+1,{{\overline{\beta}^{\,s}_{i}}}\right) E_{c\mid\mathbf{S}_{n}} \left[\left(\frac{c}{1-c}\right)^{{{\beta^{s}_{i}}}+k} I_{c<1/2}\right] + \right. \\[-2pt] & \left. \mathrm{B}\left({{\alpha^{s}_{i}}}+1,{{\overline{\alpha}^{\,s}_{i}}}\right)\mathrm{B}\left({{\beta^{s}_{i}}},{{\overline{\beta}^{\,s}_{i}}}\right)E_{c\mid\mathbf{S}_{n}} \left[ I_{c<1/2}\right] \,-\, \mathrm{B}\left({{\alpha^{s}_{i}}}+1,{{\overline{\alpha}^{\,s}_{i}}}\right) \sum_{j=0}^{{{\overline{\beta}^{\,s}_{i}}}-1} r_{j}\left({{\beta^{s}_{i}}},{{\overline{\beta}^{\,s}_{i}}}\right) E_{c\mid\mathbf{S}_{n}} \left[\left(\frac{c}{1-c}\right)^{{{\beta^{s}_{i}}}+j} I_{c<1/2}\right]\right\}\\[-2pt] &=\frac{1}{2^{\alpha^{s}}\, \mathrm{B}({\alpha^{s}},{\beta^{s}})\mathrm{B}\left({{\alpha^{s}_{i}}},{{\overline{\alpha}^{\,s}_{i}}}\right) \mathrm{B}\left({{\beta^{s}_{i}}},{{\overline{\beta}^{\,s}_{i}}}\right)}\,\times\,\\[1ex] & \times \left\{ \sum_{j=0}^{{{\overline{\alpha}^{\,s}_{i}}}-1}\:\: \sum_{k=0}^{{{\overline{\beta}^{\,s}_{i}}}-1} \:\:\sum_{l=0}^{{\beta^{s}} - \left({{\beta^{s}_{i}}}+k+1\right)} \!\! \left[ r_{j}\left({{\alpha^{s}_{i}}}+1,{{\overline{\alpha}^{\,s}_{i}}}\right)\,r_{k}\left({{\alpha^{s}_{i}}}+{{\beta^{s}_{i}}}+j+1,{{\overline{\beta}^{\,s}_{i}}}\right) r_{l}\left({\alpha^{s}}+{{\beta^{s}_{i}}}+k,{\beta^{s}} - \left({{\beta^{s}_{i}}}+k\right)\right) \right. \right. \\ & \left. \quad \quad \times\, \frac{1}{2^{{{\beta^{s}_{i}}}+k+l}}\right] +\, \mathrm{B}\left({{\alpha^{s}_{i}}}+1,{{\overline{\alpha}^{\,s}_{i}}}\right) \mathrm{B}\left({{\beta^{s}_{i}}},{{\overline{\beta}^{\,s}_{i}}}\right) \, \sum_{j=0}^{{\beta^{s}}-1} \, r_{j}\left({\alpha^{s}},{\beta^{s}}\right) \frac{1}{2^{j}} \\ & \quad\quad -\, \left. \mathrm{B}\left({{\alpha^{s}_{i}}}+1,{{\overline{\alpha}^{\,s}_{i}}}\right) \, \sum_{j=0}^{{{\overline{\beta}^{\,s}_{i}}}-1}\:\:\sum_{k=0}^{{\beta^{s}}-\left({{\beta^{s}_{i}}}+j+1\right)} \, r_{j}\left({{\beta^{s}_{i}}},{{\overline{\beta}^{\,s}_{i}}}\right) \,r_{k}\left({\alpha^{s}}+{{\beta^{s}_{i}}}+j,{\beta^{s}}-\left({{\beta^{s}_{i}}}+j\right)\right) \frac{1}{2^{{{\beta^{s}_{i}}}+j+k}} \right\}, \end{aligned}}}   $$

Likewise, we have

(12) $${\kern20pt} \begin{aligned} & E_{c\mid\mathbf{S}_{n}} \left[\frac{c}{1\,-\,c}E_{{\mathbf q}\mid\mathbf{S}_{n}} \left[E_{{\mathbf p}\mid\mathbf{S}_{n}} \left[p_{i} \,I_{p_{i} < \frac{1-c}{c} q_{i}}\right] I_{c\geq 1/2} \right] \right] \,=\, \frac{1}{2^{\beta^{s}}\,\mathrm{B}({\alpha^{s}},{\beta^{s}})\mathrm{B}\left({{\alpha^{s}_{i}}}, {{\overline{\alpha}^{\,s}_{i}}}\right) \mathrm{B}\left({{\beta^{s}_{i}}},{{\overline{\beta}^{\,s}_{i}}}\right)}\\[1ex] & \times \left\{ \sum_{j=0}^{{{\overline{\alpha}^{\,s}_{i}}}-1} \:\:\sum_{k=0}^{{\alpha^{s}} - ({{\alpha^{s}_{i}}}+j+1)} \!\! \left[r_{j}\left({{\alpha^{s}_{i}}}+1,{{\overline{\alpha}^{\,s}_{i}}}\right)\,r_{k}\left({\beta^{s}}+ {{\alpha^{s}_{i}}}+j,{\alpha^{s}}-\left({{\alpha^{s}_{i}}}+j\right)\right) \, \mathrm{B}\left({{\alpha^{s}_{i}}}+{{\beta^{s}_{i}}}+j+1,{{\overline{\beta}^{\,s}_{i}}}\right) \right. \right. \\ & \left. \left. \quad \quad \times\, \frac{1}{2^{{{\alpha^{s}_{i}}}+j+k}}\right]\vphantom{\left\{ \sum_{j=0}^{{{\overline{\alpha}^{\,s}_{i}}}-1} \:\:\sum_{k=0}^{{\alpha^{s}} - ({{\alpha^{s}_{i}}}+j+1)} \!\! \left[r_{j}\left({{\alpha^{s}_{i}}}+1,{{\overline{\alpha}^{\,s}_{i}}}\right)\,r_{k}\left({\beta^{s}}+ {{\alpha^{s}_{i}}}+j,{\alpha^{s}}-\left({{\alpha^{s}_{i}}}+j\right)\right) \, \mathrm{B}\left({{\alpha^{s}_{i}}}+{{\beta^{s}_{i}}}+j+1,{{\overline{\beta}^{\,s}_{i}}}\right) \right. \right.} \right\}, \end{aligned}   $$

(13) $$ \begin{aligned} & E_{c\mid\mathbf{S}_{n}} \left[\frac{1\,-\,c}{c}E_{{\mathbf p}\mid\mathbf{S}_{n}} \left[E_{{\mathbf q} \mid\mathbf{S}_{n}} \left[q_{i} \,I_{q_{i} \leq \frac{c}{1-c} p_{i}}\right] I_{c< 1/2} \right] \right]\\& \,=\, \frac{1}{2^{\alpha^{s}}\,\mathrm{B}({\alpha^{s}},{\beta^{s}})\mathrm{B}\left({{\alpha^{s}_{i}}},{{\overline{\alpha}^{\,s}_{i}}}\right) \mathrm{B}\left({{\beta^{s}_{i}}},{{\overline{\beta}^{\,s}_{i}}}\right)}\\[1ex] & \times \left\{ \sum_{j=0}^{{{\overline{\beta}^{\,s}_{i}}}-1} \:\:\sum_{k=0}^{{\beta^{s}} - ({{\beta^{s}_{i}}}+j+1)} \!\!\left[r_{j}\left({{\beta^{s}_{i}}}+1,{{\overline{\beta}^{\,s}_{i}}}\right)\,r_{k}({\alpha^{s}}+ {{\beta^{s}_{i}}}+j,\right.\right.\\&\quad\; \left.\left.{\beta^{s}}-({{\beta^{s}_{i}}}+j)) \, \mathrm{B}\left({{\alpha^{s}_{i}}}+ {{\beta^{s}_{i}}}+j+1,{{\overline{\alpha}^{\,s}_{i}}}\right) \right. \right. \\ & \left. \left. \quad \quad\;\; \times\, \frac{1}{2^{{{\beta^{s}_{i}}}+j+k}}\right] \vphantom{\left\{ \sum_{j=0}^{{{\overline{\beta}^{\,s}_{i}}}-1} \:\:\sum_{k=0}^{{\beta^{s}} - ({{\beta^{s}_{i}}}+j+1)} \!\!\left[r_{j}\left({{\beta^{s}_{i}}}+1,{{\overline{\beta}^{\,s}_{i}}}\right)\,r_{k}({\alpha^{s}}+ {{\beta^{s}_{i}}}+j,\right.\right.}\right\}, \end{aligned}   $$

and

(14) $${} {\fontsize{9.4pt}{9.6pt}{\begin{aligned} & E_{c\mid\mathbf{S}_{n}} \left[E_{{\mathbf p}\mid\mathbf{S}_{n}}\left[E_{{\mathbf q}\mid\mathbf{S}_{n}}\left[ q_{i} \,I_{q_{i} \leq \frac{c}{1\,-\,c} p_{i}}\right] I_{c\geq 1/2}\right] \right]\\&\,=\, \frac{1}{2^{\beta^{s}}\, \mathrm{B}({\alpha^{s}},{\beta^{s}})\mathrm{B}\left({{\alpha^{s}_{i}}},{{\overline{\alpha}^{\,s}_{i}}}\right) \mathrm{B}\left({{\beta^{s}_{i}}},{{\overline{\beta}^{\,s}_{i}}}\right)}\\[1ex] & \times \left\{ \sum_{j=0}^{{{\overline{\beta}^{\,s}_{i}}}-1}\:\: \sum_{k=0}^{{{\overline{\alpha}^{\,s}_{i}}}-1} \:\:\sum_{l=0}^{{\alpha^{s}} -({{\alpha^{s}_{i}}}+k+1)} \!\! \left[r_{j}({{\beta^{s}_{i}}}+1,{{\overline{\beta}^{\,s}_{i}}})\,r_{k}({{\alpha^{s}_{i}}}+{{\beta^{s}_{i}}}+j \right.\right.\\&\qquad \left.\left.+ \ 1,{{\overline{\alpha}^{\,s}_{i}}}) r_{l}({\beta^{s}}+{{\alpha^{s}_{i}}}+k,{\alpha^{s}} - ({{\alpha^{s}_{i}}}+k)) \right. \right. \\ & \left. \quad \quad \times\, \frac{1}{2^{{{\alpha^{s}_{i}}}+k+l}}\right] +\, \mathrm{B}({{\beta^{s}_{i}}}+1,{{\overline{\beta}^{\,s}_{i}}})\mathrm{B}({{\alpha^{s}_{i}}},{{\overline{\alpha}^{\,s}_{i}}}) \, \sum_{j=0}^{{\alpha^{s}}-1} \, r_{j}({\beta^{s}},{\alpha^{s}}) \frac{1}{2^{j}} \\ & \quad\quad -\, \left. \mathrm{B}({{\beta^{s}_{i}}}+1,{{\overline{\beta}^{\,s}_{i}}}) \, \sum_{j=0}^{{{\overline{\alpha}^{\,s}_{i}}}-1}\:\:\sum_{k=0}^{{\alpha^{s}}-({{\alpha^{s}_{i}}}+j+1)} \, r_{j}({{\alpha^{s}_{i}}},{{\overline{\alpha}^{\,s}_{i}}})\,r_{k}({\beta^{s}}+{{\alpha^{s}_{i}}}\right.\\& \qquad\left.+ \ j,{\alpha^{s}}-({{\alpha^{s}_{i}}}+j)) \frac{1}{2^{{{\alpha^{s}_{i}}}+j+k}}\vphantom{\left\{ \sum_{j=0}^{{{\overline{\beta}^{\,s}_{i}}}-1}\:\: \sum_{k=0}^{{{\overline{\alpha}^{\,s}_{i}}}-1} \:\:\sum_{l=0}^{{\alpha^{s}} -({{\alpha^{s}_{i}}}+k+1)} \!\! \left[r_{j}({{\beta^{s}_{i}}}+1,{{\overline{\beta}^{\,s}_{i}}})\,r_{k}({{\alpha^{s}_{i}}}+{{\beta^{s}_{i}}}+j \right.\right.} \right\}, \end{aligned}}}   $$

where the Beta function *B*(*a,b*) and the coefficients *r*_*i*_(*a,b*) are defined in Appendix A.

Replacing (11)–(14) into (10) produces an exact expression for computing the MMSE CoD estimator in terms of sample sizes and model hyperparameters. Notice that for the previous expressions to make sense, one must have *α*>*Δ*_*p*_−1 and *β*>*Δ*_*q*_−1. In particular, if uniform priors are chosen for **p** or **q**, then the prior for *c* cannot be uniform (c.f. Appendix A).

### Optimal Bayesian predictor CoD estimator

In this section, we derive a second Bayesian CoD estimator, using the *optimal Bayesian predictor* (OBP), a simple extension to the Boolean prediction problem of the “optimal Bayesian classifier” (OBC) proposed in [20]. Formally, let *ε*_***θ***_[*ψ*] denote the error of a predictor *ψ* under parameter vector ***θ***. The OBP predictor *ψ*_OBP_ minimizes the average error over the family of (posterior) distributions indexed by the parameter

(15) $$ \psi_{\text{OBP}} \,=\, \arg\min_{\psi\in \Upsilon} \:E_{\boldsymbol{\theta}\mid\mathbf{S}_{n}}[\!\varepsilon_{\boldsymbol{\theta}}[\!\psi]],  $$

Using the results of [20] for the OBC, one can verify that the OBP for the Beta-Dirichlet model considered here is given by

(16) $$ \psi_{\text{OBP}}(\mathbf{x}^{i}) \:=\: \left\{ \begin{array}{ll} 1, & {\textrm{if }\:\:\frac{n_{0}+\alpha}{n+\alpha+\beta} \:\frac{U_{i} + \alpha_{i}}{n_{0}+\Delta_{p}} \:<\: \frac{n_{1}+\beta}{n+\alpha+\beta} \:\frac{V_{i} + \beta_{i}}{n_{1}+\Delta_{q}}}, \\ 0, & \mathrm{otherwise,} \end{array} \right.  $$

for *i*=1,…,2^*d*^, with optimal prediction error

(17) $${} {\fontsize{9.2pt}{9.6pt}{\begin{aligned} \hat{\varepsilon}_{\text{OBP}} \,=\, E_{\boldsymbol{\theta}\mid\mathbf{S}_{n}}[\!\varepsilon_{\boldsymbol{\theta}}[\!\psi_{\text{OBP}}]] \,&=\, \sum_{i=1}^{2^{d}} \min \left\{ \frac{n_{0}+\alpha}{n+\alpha+\beta}\: \frac{U_{i} + \alpha_{i}}{n_{0}+\Delta_{p}},\right.\\&\qquad\qquad\;\;\; \left. \frac{n_{1}+\beta}{n+\alpha+\beta} \: \frac{V_{i} + \beta_{i}}{n_{1}+\Delta_{q}}\right\}. \end{aligned}}}   $$

On the other hand, the average errors of the the constant predictors *ψ*≡0 and *ψ*≡1 are

(18) $$ \begin{aligned} E_{c\mid\mathbf{S}_{n}}[\!P(Y=1)]&\,=\, E_{c\mid\mathbf{S}_{n}}[\!c] \,=\, \frac{n_{0}+\alpha}{n+\alpha+\beta}, \\ E_{c\mid\mathbf{S}_{n}}[\!P(Y=0)]&\,=\, 1-E_{c\mid\mathbf{S}_{n}}[\!c] \,=\, \frac{n_{1}+\beta}{n+\alpha+\beta}, \end{aligned}   $$

respectively, so that the OBP error in the absence of observations is

(19) $$ \hat{\varepsilon}_{0,\text{OBP}} \,=\, \min \left\{ \frac{n_{0}+\alpha}{n+\alpha+\beta},\: \frac{n_{1}+\beta}{n+\alpha+\beta} \right\},   $$

We can then combine (17) and (19) to obtain the optimal Bayesian predictor (OBP) CoD estimator

(20) $${} \begin{aligned} \widehat{\text{CoD}}_{\text{OBP}} & \,=\, 1 - \frac{\hat{\varepsilon}_{\text{OBP}}}{\hat{\varepsilon}_{0,\text{OBP}}} \\[-2ex] & \,=\, 1- \frac{1}{\min\{n_{0}+\alpha,n_{1}+\beta\}} \, \sum_{i=1}^{2^{d}} \min \\&\quad\;\;\left\{ \frac{n_{0}+\alpha}{n_{0}+\Delta_{p}} (U_{i} + \alpha_{i}),\: \frac{n_{1}+\beta}{n_{1}+\Delta_{q}} (V_{i} + \beta_{i})\right\}. \end{aligned}   $$

It is easy to show that $0 \leq \hat {\varepsilon }_{\text {OBP}} \leq \hat {\varepsilon }_{0,\text {OBP}}$, and thus $0 \leq \widehat {\text {CoD}}_{\text {OBP}} \leq 1$.

Execution time for computation of the OBP CoD estimator grows as *O*(2^*d*^). By comparison, the complexity for exact computation of the Bayesian MMSE CoD estimator introduced in the previous subsection is *O*(*n* ^3^ × 2^*d*^). Neither *n* or *d* tends to be too large in Genomics applications, due to small sample sizes and the fact that the average number of predictor genes *d* per target gene must be small for a stable system, as remarked by S. Kauffman in [2]. However, if *n* and *d* become large, one could devise Monte Carlo approximation methods to compute both CoD estimators.

Therefore, the OBP CoD estimator, though suboptimal, is much more efficient computationally than the MMSE CoD estimator, especially at large sample sizes. In addition, we will see in the next section that the OBP CoD can be even more accurate than the MMSE CoD estimator, in frequentist sense, under a fixed value of the parameters.

## Performance analysis

In this section, we investigate the accuracy of the Bayesian CoD estimators proposed in the previous section. We distinguish between two types of accuracy metrics: *global* metrics concern the average performance over all samples and all distributions of (**X,Y**), weighted by the prior distribution of ***θ***, whereas *fixed-parameter* metrics have to do with the average performance over all samples, but under a particular distribution of (**X,Y**), corresponding to a fixed value of the parameter ***θ***. Fixed-parameter metrics thus evaluate the proposed Bayesian estimators from a purely frequentist perspective.

For a given Bayesian CoD estimator, the fixed-parameter accuracy metrics of interest are the bias

(21) $$ \text{Bias}(\boldsymbol{\theta}) \,=\,E_{\mathbf{S}_{n} \mid \boldsymbol{\theta}}\!\left[\widehat{\text{CoD}} - \text{CoD}\right] \,=\, E_{\mathbf{S}_{n} \mid \boldsymbol{\theta}}\!\left[\frac{\hat{\varepsilon}}{\hat{\varepsilon}_{0}} \right] -\frac{\varepsilon}{\varepsilon_{0}},  $$

the variance,

(22) $${} {\fontsize{8.8pt}{9.6pt}{\begin{aligned} \text{Variance}(\boldsymbol{\theta}) \,=\, \text{Var}_{\mathbf{S}_{n} \mid \boldsymbol{\theta}}\!\left[\widehat{\text{CoD}}\right] \,=\, E_{\mathbf{S}_{n} \mid \boldsymbol{\theta}}\!\left[\:\frac{\hat{\varepsilon}^{2}}{\hat{\varepsilon}_{0}^{2}}\:\right] - \left(E_{\mathbf{S}_{n} \mid \boldsymbol{\theta}}\!\left[ \frac{\hat{\varepsilon}}{\hat{\varepsilon}_{0}} \right] \right)^{2}, \end{aligned}}}  $$

and the root-mean-square (RMS) error,

(23) $$\begin{array}{@{}rcl@{}} \text{RMS}(\boldsymbol{\theta}) \,&=&\, \sqrt{E_{\mathbf{S}_{n} \mid \boldsymbol{\theta}}\!\left[\left(\widehat{\text{CoD}}-\text{CoD}\right)^{2}\right]} \notag\\ &=&\, \sqrt{\:\text{Variance}(\boldsymbol{\theta}) \,+\, \text{Bias}(\boldsymbol{\theta})^{2}}, \end{array} $$

It becomes clear that the fixed-parameter bias, variance, and RMS of a Bayesian CoD estimator can be obtained with knowledge of the first and second moments $E_{\mathbf {S}_{n} \mid \boldsymbol {\theta }}\!\left [ \frac {\hat {\varepsilon }}{\hat {\varepsilon }_{0}} \right ]$ and $E_{\mathbf {S}_{n} \mid \boldsymbol {\theta }}\!\left [\frac {\hat {\varepsilon }^{2}}{\hat {\varepsilon }_{0}^{2}}\right ]$.

The corresponding global accuracy metrics are obtained by taking expectation of the previous quantities with respect to the marginal (i.e., prior) distribution of ***θ***.

As mentioned previously, the global bias of the Bayesian MMSE CoD estimator is zero and its global RMS is minimal among all CoD estimators. However, this does not imply that its fixed-parameter bias is zero or that its fixed-parameter RMS is minimum for all values of the parameter.

In what follows, we give exact expressions for the computation of $E_{\mathbf {S}_{n} \mid \boldsymbol {\theta }}\!\left [ \frac {\hat {\varepsilon }}{\hat {\varepsilon }_{0}} \right ]$ and $E_{\mathbf {S}_{n} \mid \boldsymbol {\theta }}\!\left [\frac {\hat {\varepsilon }^{2}} {\hat {\varepsilon }_{0}^{2}}\right ]$for the OBP CoD estimator. As argued previously, this allows the exact computation of the fixed-parameter bias, variance, and RMS of that CoD estimator. Via simple numerical integration, it is possible then to obtain the global bias, variance, and RMS. It turns out that similar expressions for the MMSE CoD estimator are much harder to obtain; the performance of that estimator are studied via a numerical approach in the next section.

All the expectations and probabilities below are with respect to **S**_*n*_ ∣ ***θ*** (the subscript will be omitted for convenience). In the expressions below, *c*, *p*_*i*_, and *q*_*i*_, for *i*=1,…,2^*d*^ refer to the (deterministic) parameters in ***θ***.

First note that

(24) $${} {\fontsize{9.2pt}{9.6pt}{\begin{aligned} E\left[\frac{\hat{\varepsilon}_{\text{OBP}}}{\hat{\varepsilon}_{0,\text{OBP}}} \right] \:&=\:E\left[E\left[\frac{\hat{\varepsilon}_{\text{OBP}}}{\hat{\varepsilon}_{0,\text{OBP}}} \;\bigg|\;\hat{\varepsilon}_{0,\text{OBP}}\right]\right] \\ &= \: \sum_{m \in L} E\left[\frac{\hat{\varepsilon}_{\text{OBP}}}{m/(n+\alpha+\beta)} \;\bigg|\; M = m \right] P(M = m), \end{aligned}}}   $$

where $M = (n+\alpha +\beta)\,\hat {\varepsilon }_{0,\text {OBP}} = \min (n_{0}+\alpha, n_{1}+\beta)$ and

$$\begin{aligned} L \,&=\, \left \{\alpha, \alpha+1, \ldots, \alpha + \Big\lfloor \frac{n+\beta-\alpha}{2} \Big\rfloor \right\} \bigcup \\&\quad\;\, \left \{ \beta, \beta+1, \ldots, \beta + \Big\lfloor \frac{n+\alpha-\beta}{2} \Big\rfloor \right \}, \end{aligned} $$

where ⌊*x*⌋ denotes that the largest integer smaller or equal to *x*. Let $L_{0} = \left \{\alpha, \alpha +1, \ldots, \lfloor \frac {n+\beta -\alpha }{2} \rfloor + \alpha \right \}$, $L_{1} = \left \{\beta, \beta +1, \ldots, \lfloor \frac {n+\alpha -\beta }{2} \rfloor + \beta \right \}$. There are three possibilities: (1) *α*−⌊*α*⌋≠*β*−⌊*β*⌋; (2) *α*−⌊*α*⌋=*β*−⌊*β*⌋ and *α*=*β*; (3) *α*−⌊*α*⌋=*β*−⌊*β*⌋ but *α*≠*β*. We will provide the derivation only in case (3); the other cases are similar, and lead to the exact same expressions.

We assume that *α*−⌊*α*⌋=*β*−⌊*β*⌋ but *α*≠*β*. Without loss of generality, we assume that *α*>*β*, and let *α*=*β*+*δ*, where *δ* is a positive integer. Notice that it is easy to show in this case that $\lfloor \frac {n+\beta -\alpha }{2} \rfloor + \alpha = \lfloor \frac {n+\alpha -\beta }{2} \rfloor + \beta $ by considering the evenness and oddness of *n* and *δ*. Therefore, we have *L*_0_⊂*L*_1_. In the following, we discuss two cases when *n*+*β*−*α* is even and when *n*+*β*−*α* is odd.

(1) When *n*+*β*−*α* is even, the event [*M*=*m*] is equal to the union of the disjoint events [ *n*_0_=*m*−*α*], and [*n*_1_=*m*−*β*]=[*n*_0_=*n*−*m*+*β*], for $m\in L_{0} \backslash \left \{\alpha + \frac {n+\beta -\alpha }{2} \right \}$, whereas $\left [M=\alpha +\frac {n+\beta -\alpha }{2} \right ] = \left [n_{0} = m - \alpha = \frac {n+\beta -\alpha }{2} \right ]$, and [ *M*=*m*] = [ *n*_0_=*n*−*m*+*β*], for *m*∈*L*_1_∖*L*_0_.

Now, we are going to use the fact that, for a random variable *X* and disjoint events *A* and *B*, one has^1^

(25) $${\kern20pt} \begin{aligned} E\left[X \mid A \cup B\right] \:=\: \frac{P(A)}{P(A) \:+\: P(B)}\,E\left[X \mid A\right] \:+\: \frac{P(B)}{P(A)+P(B)}\,E[\!X \mid B], \end{aligned}   $$

We can then write $E\left [ \frac {\hat {\varepsilon }_{\text {OBP}}}{\hat {\varepsilon }_{0,\text {OBP}}} \right ]$ as:

(26) $${\kern20pt} \begin{aligned} E\left[\frac{\hat{\varepsilon}_{\text{OBP}}}{\hat{\varepsilon}_{0,\text{OBP}}} \right] \:=\: & \sum_{m\in L_{0} \backslash \left \{ \frac{n+\Delta_{c}}{2} \right \}} E\left[\frac{\hat{\varepsilon}_{\text{OBP}}}{m/(n+\Delta_{c})} \;\bigg|\; n_{0} = m - \alpha\right]\! P(n_{0} = m - \alpha) \,\,+\,\, \\ & \sum_{m\in L_{0} \backslash \left\{ \frac{n+\Delta_{c}}{2} \right \}} E\left[\frac{\hat{\varepsilon}_{\text{OBP}}}{m/(n+\Delta_{c})} \;\bigg|\; n_{0} =n\,-\,m + \beta\right]\! P(n_{0} = n\,-\,m + \beta) \,\,+\,\, \\ & \sum_{m\in L_{1}\backslash L_{0}} E\left[\frac{\hat{\varepsilon}_{\text{OBP}}}{m/(n+\Delta_{c})} \;\bigg|\; n_{0} =n\,-\,m + \beta\right]\! P(n_{0} = n\,-\,m + \beta) \,\,+\,\, \\ & E\left[\frac{\hat{\varepsilon}_{\text{OBP}}}{m/(n+\Delta_{c})} \;\bigg|\; n_{0} = \frac{n-\alpha +\beta}{2}\right]\! P\left(n_{0} = \frac{n-\alpha +\beta}{2} \right) \\ \:=\:& \sum_{m\in L_{0}} E\left[\frac{\hat{\varepsilon}_{\text{OBP}}}{m/(n+\Delta_{c})} \;\bigg|\; n_{0} = m - \alpha\right]\! P(n_{0} = m - \alpha) \,\,+\,\, \\ & \sum_{m\in L_{1}} E\left[\frac{\hat{\varepsilon}_{\text{OBP}}}{m/(n+\Delta_{c})} \;\bigg|\; n_{0} =n\,-\,m + \beta\right]\! P(n_{0} = n\,-\,m + \beta) I_{m \neq \frac{n+\alpha +\beta}{2}} \\ \:=\: & \sum_{r=0}^{\lfloor \frac{n+\beta-\alpha}{2} \rfloor} E\left[\frac{\hat{\varepsilon}_{\text{OBP}}}{(n_{0}+\alpha)/(n+\Delta_{c})} \;\bigg|\; n_{0} = r\right]\! P(n_{0} = r) \,\,+\,\, \\ & \sum_{r=0}^{\lfloor \frac{n+\alpha-\beta}{2} \rfloor} E\left[\frac{\hat{\varepsilon}_{\text{OBP}}}{(n_{1}+\beta)/(n+\Delta_{c})} \;\bigg|\; n_{0} =n\,-\,r\right]\! P(n_{0} = n\,-\,r) I_{r \,\neq \frac{n+\alpha-\beta}{2}} \\ \end{aligned}   $$

where $P(n_{0} = r) \,=\, \binom {n}{r}c^{r}(1-c)^{n-r}$ and

(27) $${\kern20pt} \begin{aligned} & E\left[\frac{\hat{\varepsilon}_{\text{OBP}}}{(n_{0}+\alpha)/(n+\Delta_{c})} \;\bigg|\; n_{0} = r\right] \\ & \,=\, \sum_{i=1}^{2^{d}} \left \{\sum_{\substack{\frac{(r+\alpha)(k+\alpha_{i})}{r+\Delta_{p}} < \frac{(n-r+\beta)(l+\beta_{i})}{n-r+\Delta_{q}} \\ k \leq r,\,\, k+l \leq n}}\: \frac{(r+\alpha)(k+\alpha_{i})}{r+\Delta_{p}} P(U_{i}=k,V_{i}=l\mid n_{0} = r) \right. \\ & \hspace{0.0em} \left. \quad\,+\, \sum_{\substack{\frac{(r+\alpha)(k+\alpha_{i})}{r+\Delta_{p}} \geq \frac{(n-r+\beta)(l+\beta_{i})}{n-r+\Delta_{q}} \\ k \leq r, \,\,k+l \leq n}}\: \frac{(n-r+\beta)(l+\beta_{i})}{n-r+\Delta_{q}}P(U_{i}=k,V_{i}=l\mid n_{0} = r)\right \}, \end{aligned}   $$

with $P(U_{i}=k,V_{i}=l \mid n_{0} = r) \,=\, \binom {r}{k}{p_{i}^{k}}(1-p_{i})^{r-k}\binom {n-r}{l}{q_{i}^{l}}(1-q_{i})^{n-r-l}$. The expression for $E\left [\frac {\hat {\varepsilon }_{\text {OBP}}}{(n_{1}+\beta)/(n+\Delta _{c})} \;\big |\; n_{0} = n-r\right ]$ is obtained from (27), with *r*+*α* replaced by *r*+*β*.

(2) When *n*+*β*−*α* is odd, the event [*M*=*m*] is equal to the union of the disjoint events [*n*_0_=*m*−*α*], and [*n*_1_=*m*−*β*]=[*n*_0_=*n*−*m*+*β*], for *m*∈*L*_0_, whereas [*M*=*m*]=[*n*_0_=*n*−*m*+*β*], for *m*∈*L*_1_∖*L*_0_. By applying the same reasoning, we have the same expression as in (26). Note that $I_{n_{1} \neq \frac {n+\alpha -\beta }{2}} $ is always equal to 1 in this case since $\frac {n+\alpha -\beta }{2}$ is not an integer.

For the second moment, we have

(28) $${\kern20pt} E\left[\frac{\hat{\varepsilon}_{\text{OBP}}^{2}}{\hat{\varepsilon}_{0,\text{OBP}}^{2}} \right] \:=\:E\left[E\left[\frac{\hat{\varepsilon}_{\text{OBP}}^{2}}{\hat{\varepsilon}_{0,\text{OBP}}^{2}} \;\bigg|\;\hat{\varepsilon}_{0,\text{OBP}}\right]\right] \: = \: \sum_{m \in L} E\left[\left(\frac{\hat{\varepsilon}_{\text{OBP}}}{m/(n+\Delta_{c})} \right)^{2} \,\bigg|\; M = m \right] P(M = m),   $$

where $M = (n+\Delta _{c})\,\hat {\varepsilon }_{0,\text {OBP}}$, as before. By using the same reasoning applied previously in the case of the first moment, we have

(29) $${} {\fontsize{9.2pt}{9.6pt}{\begin{aligned} E\left[\frac{\hat{\varepsilon}_{\text{OBP}}^{2}}{\hat{\varepsilon}_{0,\text{OBP}}^{2}} \right] \:=\: &\sum_{m\in L_{0}} E\left[\frac{\hat{\varepsilon}_{\text{OBP}}^{2}}{m^{2}/(n+\Delta_{c})^{2}} \;\bigg|\; n_{0} = m - \alpha\right]\! \\& P(n_{0} = m - \alpha) \,\,+\,\, \\ & \sum_{m\in L_{1}} E\left[\frac{\hat{\varepsilon}_{\text{OBP}}^{2}}{m^{2}/(n+\Delta_{c})^{2}} \;\bigg|\; n_{0} =n\,-\,m + \beta\right]\! \\& P(n_{0} = n\,-\,m + \beta) I_{m \neq \frac{n+\alpha+\beta}{2}}, \\ \:=\: & \sum_{t=0}^{\lfloor \frac{n+\beta-\alpha}{2} \rfloor} E\left[\frac{\hat{\varepsilon}_{\text{OBP}}^{2}}{(n_{0}+\alpha)^{2}/(n+\Delta_{c})^{2}} \;\bigg|\; n_{0} = t\right]\! \\& P(n_{0} = t) \,\,+\,\, \\ & \sum_{t=0}^{\lfloor \frac{n+\alpha-\beta}{2} \rfloor} E\left[\frac{\hat{\varepsilon}_{\text{OBP}}^{2}}{(n_{1}+\beta)^{2}/(n+\Delta_{c})^{2}} \;\bigg|\; n_{0} =n\,-\,t\right]\!\\& P(n_{0} = n\,-\,t) I_{t \neq \frac{n+\alpha-\beta}{2}} \end{aligned}}}   $$

where, letting $k^{\prime }_{i} = \frac {(r+\alpha)(k+\alpha _{i})}{r+\alpha _{i}}$, $l^{\prime }_{i} = \frac {(n-r+\alpha)(l+\beta _{i})}{n-r+\beta _{i}}$, $r^{\prime }_{j} = \frac {(r+\alpha)(r+\alpha _{i})}{r+\alpha _{j}}$, $s^{\prime }_{j} = \frac {(n-r+\alpha)(s+\beta _{i})}{n-r+\beta _{j}}$, for *i,j*=1,…,*b*, we have

(30) $${} \begin{aligned} & E\left[\frac{\hat{\varepsilon}_{\text{OBP}}^{2}}{(n_{0}+\alpha)^{2}/(n+\Delta_{c})^{2}} \;\bigg|\; n_{0} = t\right]\:=\: \frac{1}{(t+\alpha)^{2}} \:\times\: \\ & \sum_{i=1}^{2^{d}} \left\{ \sum_{l^{\prime}_{i}>k^{\prime}_{i}}k^{\prime 2}_{j} P(U_{i}=k^{\prime}_{i}, V_{i}=l^{\prime}_{i} \mid n_{0}=t) \,\right.\\&\qquad + \left. \sum_{k \geq l} l^{\prime 2}_{i} P(U_{i}=k'_{i}, V_{i}=l'_{i} \mid n_{0}=t) \right \} \:+ \\ & \frac{1}{(t+\alpha)^{2}} \sum_{\substack{i,j=1 \\ i \neq j}}^{2^{d}} \left\{ \sum_{l'_{i}>k'_{i}}\sum_{s'_{j}>r'_{j}} k'_{i} r'_{j} P(U_{i}=k'_{i}, V_{i}=l'_{i}, U_{j}=r'_{j},\right.\\&\qquad\qquad\quad\qquad \left. V_{j}=s'_{j} \mid n_{0}=t) \:+ \right.\\[-1ex] & \hspace{7.2em} \left.\sum_{l'_{i}>k'_{i}} \sum_{r'_{j}\geq s'_{j}} k'_{i} s'_{j} P(U_{i}=k'_{i}, V_{i}=l'_{i}, U_{j}=r'_{j},\right.\\[-1.5ex]& \hspace{7.2em}\left. V_{j}=s'_{j} \mid n_{0}=t) \: +\right. \\[-1ex] & \hspace{7.2em}\left. \sum_{k'_{i}\geq l'_{i}} \sum_{s'_{j}>r'_{j}} l'_{i} r'_{j} P(U_{i}=k'_{i}, V_{i}=l'_{i}, U_{j}=r'_{j},\right.\\[-1.5ex] & \hspace{7.2em}\left. V_{j}=s'_{j} \mid n_{0}=t) \:\right.+ \\[-1ex] & \hspace{7.2em} \left. \sum_{k'_{i} \geq l'_{i}} \sum_{r'_{j} \geq s'_{j}} l'_{i} s'_{j} P(U_{i}=k'_{i}, V_{i}=l'_{i}, U_{j}=r'_{j},\right.\\[-1.5ex] & \hspace{7.2em}\left. V_{j}=s'_{j} \mid n_{0}=t)\vphantom{\left\{ \sum_{l'_{i}>k'_{i}}\sum_{s'_{j}>r'_{j}} k'_{i} r'_{j} P(U_{i}=k'_{i}, V_{i}=l'_{i}, U_{j}=r'_{j},\right.} \right\}, \end{aligned}   $$

with $P(U_{i}=k, V_{i}=l, U_{j}=r, V_{j}=s \mid n_{0}=t) = \binom {n_{0}}{k,r}{p_{i}^{k}}{p_{j}^{r}}(1-p_{i}-p_{j})^{n_{0}-k-r} \binom {n-n_{0}}{l,s}{q_{i}^{l}}{q_{j}^{s}}(1-q_{i}-q_{j})^{n-n_{0}-l-s}$. The expression for $E\left [\frac {\hat {\varepsilon }_{\text {OBP}}^{2}}{(n_{1}+\beta)^{2}/(n+\Delta _{c})^{2}} \;\bigg |\; n_{0} = n - t\right ]$ is obtained from (30) with *t*+*α* replaced by *t*+*β*.

## Numerical experiments

### Global accuracy

In this section, we employ Monte Carlo sampling (with *M*=10,000 simulated data sets for each sample size) to compute global accuracy metrics of the two Bayesian CoD estimators. Following [17], we let *α*=2^*d*^+1=*β*=2^*d*^+1, which produces a prior for *c* peaked around the value *c*=0.5, and *α*_*i*_=*β*_*i*_=1, for all *i*=1,…,2^*d*^, i.e. flat (uniform) prior distributions for (**p**,**q**). In each iteration, the values of *c* and (**p**,**q**) are drawn from the respective priors, and then sample data is generated according to these probabilities. Given the sample data, we compute the exact Bayesian MMSE and OBP CoD estimates as expressed in Section 3, and compare them to the standard resubstitution CoD estimator, which is based on plugging in sample frequencies in the expression for the optimal CoD, and corresponds to the the original choice of CoD estimator in [7]. This estimator is also called the nonparametric maximum-likelihood CoD estimator in [15]. For further comparison, we also compute CoD estimators based on leave-one-out, 0.632 bootstrap and 10-repeated twofold cross-validation error estimators—for details on all these CoD estimators, please see [14, 15]. Sample means and sample variances are employed to approximate the global accuracy metrics of each CoD estimator.

**Fig. 1:**
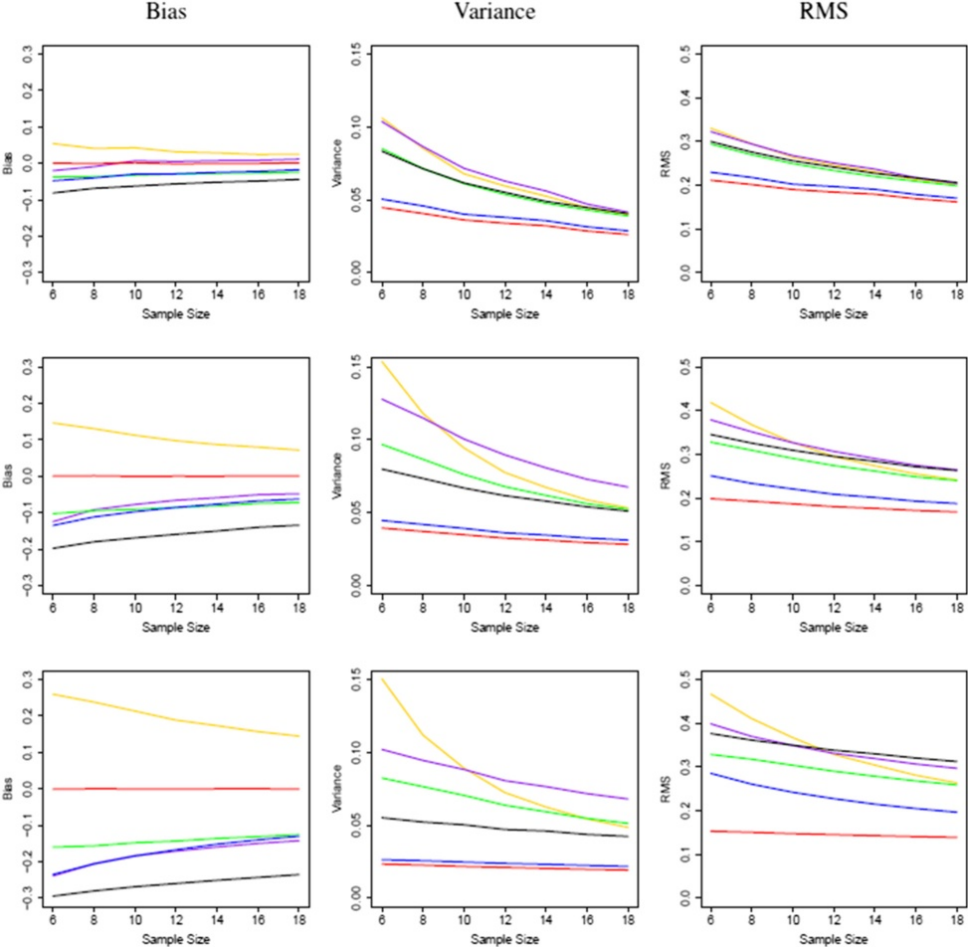
Global bias, variance, and RMS for several CoD estimators versus sample size *n*, for different numbers of predicting binary variables *d*. *Top row*: *d*=1; *Middle row*: *d*=2; *Bottom row*: *d*=3. Plot key: MMSE (*red*), OBP (*blue*), resubstitution (*gold*), leave-one-out (*purple*), 0.632 bootstrap (*green*), 10-repeated twofold cross-validation (*black*)

Figure 1 displays the global bias, variance, and RMS as a function of varying sample size, for different numbers of binary predictive variables, *d*=1 through *d*=3. Several observations are evident. First, as expected, the Bayesian MMSE CoD estimator is unbiased and has the least RMS among all the estimators, and the gap in performance widens as dimensionality increases. Secondly, the OBP CoD estimator has the second-best performance, which indicates the benefits of using the Bayesian estimation approach. The accuracy of the OBP CoD estimator is quite close to that of the MMSE estimator for *d*=1, but the gap widens as *d* increases. Thirdly, it is also observed that the OBP Bayesian CoD estimator is pessimistically biased. Incidentally, the 0.632 bootstrap CoD estimator displays the best accuracy among the four nonparametric ones according to global RMS, but it is matched by the resubstitution CoD estimator as sample size increases.

### Fixed-parameter accuracy

In this section, we study the average accuracy of the two proposed Bayesian CoD estimators for a fixed parameter, that is, we evaluate the Bayesian estimators from a purely frequentist perspective.

**Fig. 2:**
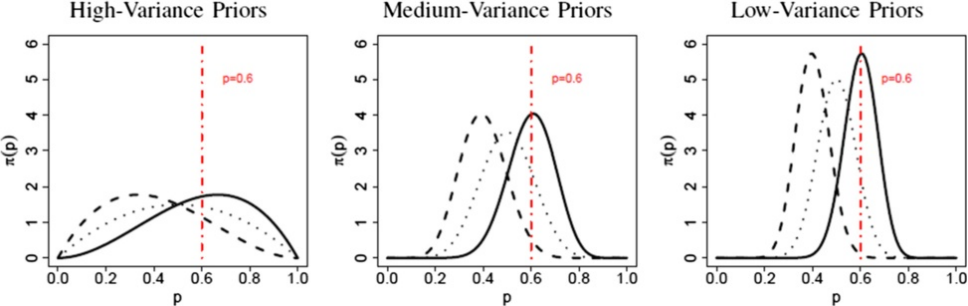
Beta prior densities for *p*_1_ and *d*=1, for different values of the concentration parameter: *Δ*_*p*_=5 (high-variance priors), *Δ*_*p*_=25 (medium-variance priors), and *Δ*_*p*_=50 (low-variance priors). Legend: *dashed line* (mismatched prior), *dotted line* (poorly matched prior), and *solid line* (matched prior). The base measures are given in Table 1, and the true value of the parameter is indicated by a *vertical line*

As in the previous subsection, we consider *d*=1 through *d*=3 binary predictive variables. We consider fixed values of the parameters, *c* ^∗^, **p** ^∗^, and **q** ^∗^. In order to examine the effect of prior belief on performance, we consider four scenarios regarding prior density around the true parameters: a flat (“non-informative”) prior and three nonflat “matched,” “poorly matched,” and “mismatched” priors. This is done by assuming different base measures and concentration parameters for the priors (c.f. Section 3). As an illustration of the approach, consider the case *d*=1. In our simulation, *c* ^∗^=0.5, **p** ^∗^=(0.6,0.4), and **q** ^∗^=(0.4,0.6), and the base measures for the nonflat priors are *c*_0_=0.5, **p**_0_=**p** ^∗^, **q**_0_=**q** ^∗^ (matched prior), *c*_0_=0.5, **p**_0_=(0.5,0.5), **q**_0_=(0.5,0.5) (poorly matched prior), and *c*_0_=0.5, **p**_0_=(0.4,0.6), **q**_0_=(0.6,0.4) (mismatched prior). In addition, we consider different values of the concentration parameters to reflect different degrees of peaking of the prior distributions. Figure 2 plots the nonflat prior densities for *p*_1_ (which is Beta-distributed), for different values of the concentration parameter: *Δ*_*p*_=5 (high-variance), *Δ*_*p*_=25 (medium variance), and *Δ*_*p*_=50 (low variance). Notice that each density is centered around the expected value. Note that, if the variance is high, even the matched prior becomes very diffuse around its expected value (which is the true value, in this case).

**Table 1:** True distributions and nonflat prior base measures for fixed-parameter experiments. In all cases, *c* ^∗^=*c*_0_=0.5, and **q** ^∗^ and **q**_0_ are obtained from **p** ^∗^ and **p**_0_, respectively, by flipping left to right (see text.)

	True distribution	Base measure 1	Base measure 2	Base measure 3
*d*=1	**p** ^∗^ = (0.6,0.4)	${\mathbf p}_{0}^{1} \,=\, (0.6,0.4)$	${\mathbf p}_{0}^{2} \,=\, (0.5,0.5)$	${\mathbf p}_{0}^{3} \,=\, (0.4,0.6)$
*d*=2	**p** ^∗^ = (0.2,0.3,0.1,0.4)	${\mathbf p}_{0}^{1} \,=\, (0.2,0.3,0.1,0.4)$	${\mathbf p}_{0}^{2} \,=\, (0.3,0.2,0.2,0.3)$	${\mathbf p}_{0}^{3} \,=\, (0.4,0.1,0.3,0.2)$
*d*=3	**p** ^∗^ = (0.1,0.15,0.05,0.2,	${\mathbf p}_{0}^{1} \,=\, (0.1,0.15,0.05,0.2,$	${\mathbf p}_{0}^{2} \,=\, (0.15, 0.1, 0.1, 0.15,$	${\mathbf p}_{0}^{3} \,=\, (0.2, 0.05, 0.15, 0.1,$
	0.15,0.1,0.1,0.15)	0.15,0.1,0.1,0.15)	0.1,0.05,0.15,0.2)	0.05,0.2,0.2,0.05)
		Matched prior	Poorly matched prior	Mismatched prior

Table 1 gives the values of the parameters used in the experiments. In all cases, the true value and base measure for *c* are the same, *c* ^∗^=*c*_0_=0.5. In addition, in each case, the true value **q** ^∗^ and base measure **q**_0_ are obtained from **p** ^∗^ and **p**_0_, respectively, by flipping the corresponding vector left to right; for example, when **p**_0_=(0.2,0.1,0.3,0.4) then **q**_0_=(0.4,0.3,0.1,0.2). Therefore, only the values for **p** ^∗^ and **p**_0_ are shown in Table 1.

**Fig. 3:**
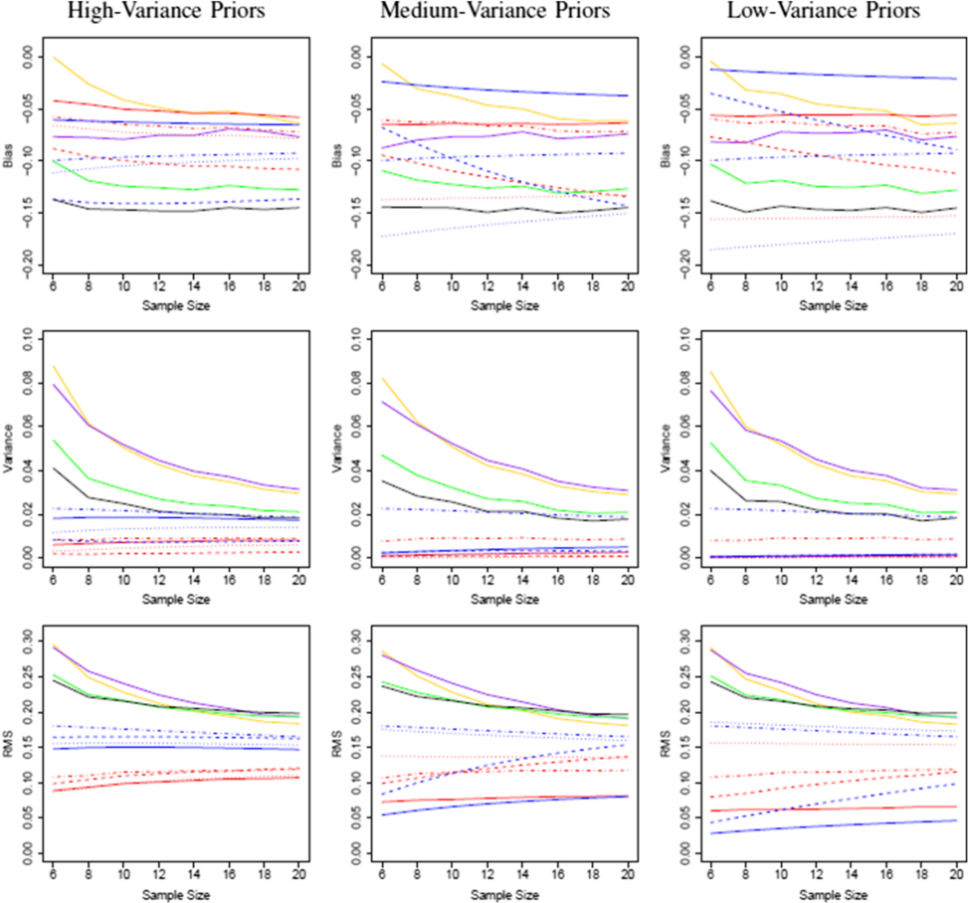
Bias, variance, and RMS for several CoD estimators versus sample size for *d*=1, under different values of the concentration parameter: *Δ*_*c*_/2=*Δ*_*p*_=*Δ*_*q*_=5 (high-variance priors), *Δ*_*c*_/2=*Δ*_*p*_=*Δ*_*q*_=25 (medium-variance priors), and *Δ*_*c*_/2=*Δ*_*p*_=*Δ*_*q*_=50 (low-variance priors). Plot key for base measures: *dash-dot line* (uniform prior), *dashed line* (mismatched prior), *dotted line* (poorly matched prior), and *solid line* (matched prior); see Table 1. Plot key for CoD estimators: MMSE (*red*), OBP (*blue*), resubstitution (*gold*), leave-one-out (*purple*), 0.632 bootstrap (*green*), 10-repeated twofold cross-validation (*black*). Results for the OBP CoD estimator are exact while others are approximated by Monte Carlo sampling method. The curves for the nonparametric CoD estimators and the flat prior Bayesian CoD estimators are repeated across the columns, for comparison with the results for the nonflat prior Bayesian CoD estimators

**Fig. 4:**
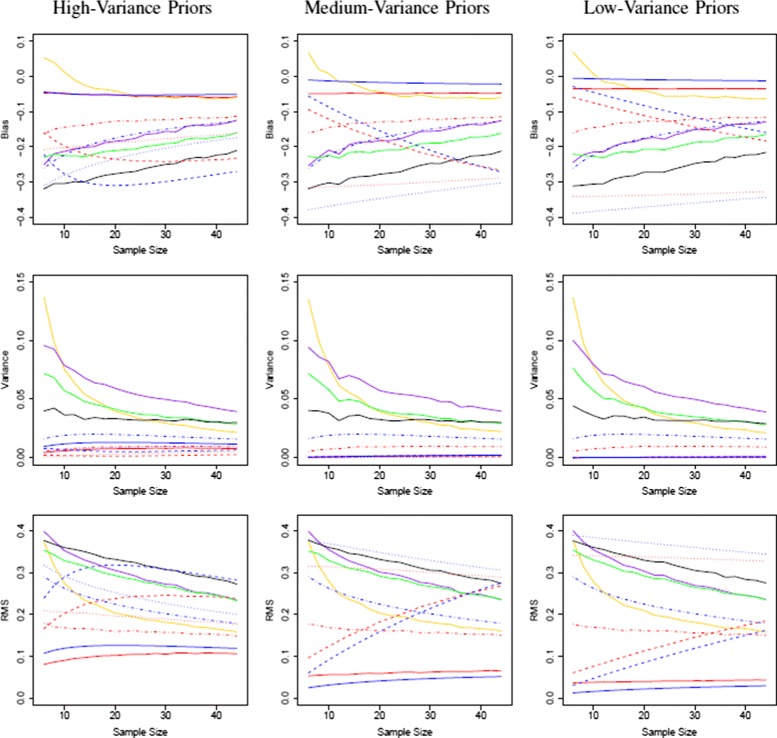
Bias, variance, and RMS for several CoD estimators versus sample size for *d*=2, under different values of the concentration parameter: *Δ*_*c*_/2=*Δ*_*p*_=*Δ*_*q*_=10 (high-variance priors), *Δ*_*c*_/2=*Δ*_*p*_=*Δ*_*q*_=50 (medium-variance priors), and *Δ*_*c*_/2=*Δ*_*p*_=*Δ*_*q*_=100 (low-variance priors). Plot key for base measures: *dash-dot line* (uniform prior), *dashed line* (mismatched prior), *dotted line* (poorly matched prior), and *solid line* (matched prior); see Table 1. Plot key for CoD estimators: MMSE (*red*), OBP (*blue*), resubstitution (*gold*), leave-one-out (*purple*), 0.632 bootstrap (*green*), 10-repeated twofold cross-validation (*black*). Results for the OBP CoD estimator are exact while others are approximated by Monte Carlo sampling method. The curves for the nonparametric CoD estimators and the flat prior Bayesian CoD estimators are repeated across the columns, for comparison with the results for the nonflat prior Bayesian CoD estimators

**Fig. 5:**
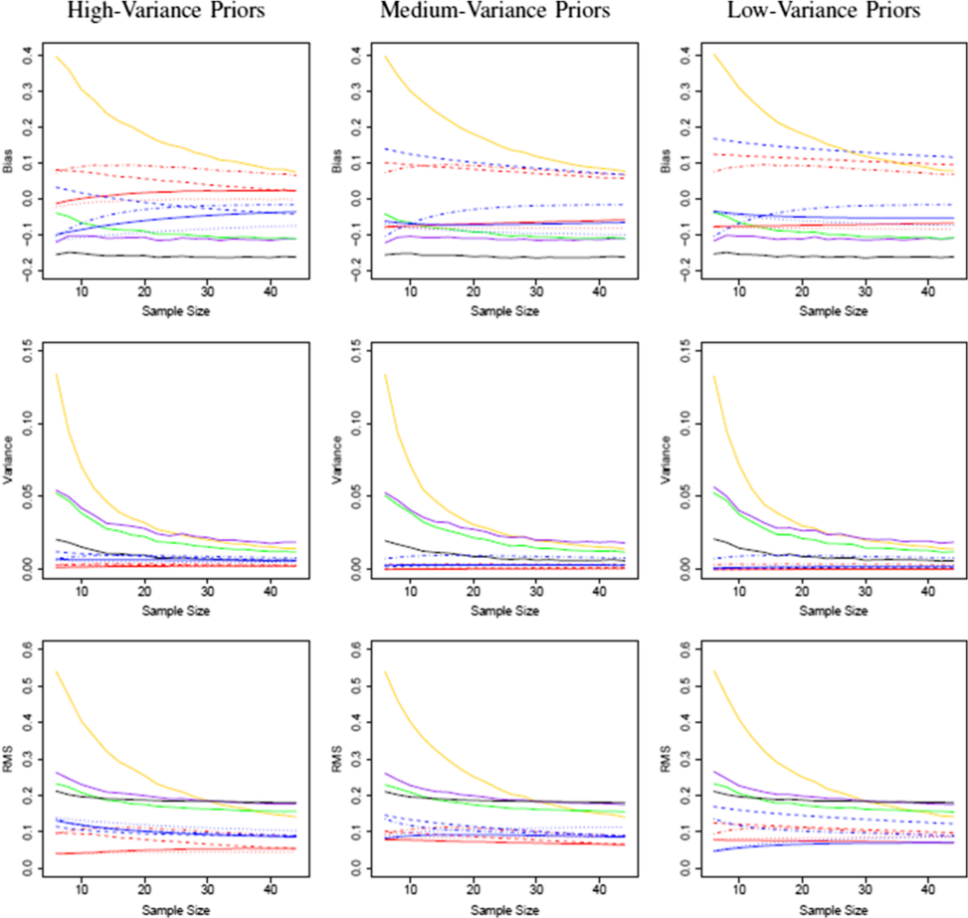
Bias, variance, and RMS for several CoD estimators versus sample size for *d*=3, under different values of the concentration parameter: *Δ*_*c*_/2=*Δ*_*p*_=*Δ*_*q*_=20 (high-variance priors), *Δ*_*c*_/2=*Δ*_*p*_=*Δ*_*q*_=100 (medium-variance priors), and *Δ*_*c*_/2=*Δ*_*p*_=*Δ*_*q*_=200 (low-variance priors). Plot key for base measures: *dash-dot line* (uniform prior), *dashed line* (mismatched prior), *dotted line* (poorly matched prior), and *solid line* (matched prior); see Table 1. Plot key for CoD estimators: MMSE (*red*), OBP (*blue*), resubstitution (*gold*), leave-one-out (*purple*), 0.632 bootstrap (*green*), 10-repeated twofold cross-validation (*black*). Results for the OBP CoD estimator are exact while others are approximated by Monte Carlo sampling method. The curves for the nonparametric CoD estimators and the flat prior Bayesian CoD estimators are repeated across the columns, for comparison with the results for the nonflat prior Bayesian CoD estimators

Figures 3, 4, and 5 show the results for *d*=1 through *d*=3 predictors, respectively. Each figure displays the bias, variance, and RMS as a function of the sample size for the Bayesian MMSE and OBP CoD estimators and the nonparametric CoD estimators. The Bayesian estimators assume a flat non-informative prior and three nonflat matched, poorly matched, and mismatched priors, specified by Table 1. For the non-flat priors only, three different variance groups are considered, corresponding to three different settings for the concentration parameters: high variance, medium variance, and low variance priors. Results for the OBP CoD estimator are computed exactly using the results of Section 4. For all other CoD estimators, bias, variance, and RMS are approximated by averaging results over 5000 Monte Carlo samples drawn from the fixed distribution. The curves for the nonparametric CoD estimators and the flat prior Bayesian CoD estimators are repeated across the columns, for comparison with the results for the nonflat prior Bayesian CoD estimators.

We can observe that, as expected, both Bayesian CoD estimators perform better when the prior is matched to the true value of the parameters than when the match is poor or nonexistent. In addition, for the matched prior, accuracy improves substantially as one moves from a diffuse (high-variance) to a peaked (low-variance) prior. This effect is especially visible in the case of the OBP CoD estimator. For example, with *d*=1 the RMS is reduced by nearly 80 % between the high-variance and low-variance matched priors. In fact, the accuracy of the OBP CoD estimator beats that of the MMSE CoD estimator for peaked priors, while the opposite is true under diffuse priors. Both Bayesian CoD estimators outperform the nonparametric ones in cases *d*=1 and *d*=3, whereas, in the *d*=2 case, the Bayesian estimators based on mismatched or poorly matched priors can perform worse than the nonparametric estimators, for larger sample size. It is also observed that, as the variance of priors decreases (i.e., for a larger *δ* value), the performance of both Bayesian estimators improves over the nonparametric ones. Moreover, it is interesting that the Bayesian MMSE CoD estimator performs better than the OBP CoD estimator, for a high-variance prior with matched prior, while the OBP CoD estimator beats the Bayesian MMSE CoD estimator for medium and high-variance matched priors. This indicates that the OBP CoD estimator is preferable due to its straightforward representation and superior performance with low-variance priors. Notice that the Bayesian MMSE CoD estimator has the least RMS only when averaged over all distributions and all possible samples, but its optimality does not apply to the settings with a fixed distribution. In addition, we observe that the Bayesian MMSE CoD estimator is less variant than the OBP CoD estimator. It can be seen that the Bayesian CoD estimators based on informative priors are less variant than those based on non-informative uniform priors. In the *d*=1 and *d*=3 cases, the OBP CoD estimator with uniform priors becomes more variant than even the cross-validation estimator, for larger sample size. In addition, the OBP CoD estimator is less biased in magnitude than the MMSE estimator for low-variance matched priors. However, as the variance of priors increases, the Bayesian MMSE CoD estimator turns out to have less bias than the OBP estimator.

## Gene regulatory network inference: a melanoma example

We discuss in this section the application of the Bayesian CoD estimation approach discussed previously to the inference of gene regulatory networks. We apply the proposed inference procedure on data collected in a study of metastatic melanoma [24], containing 31 binarized sample expression profiles, which have been binarized, with 0 indicate no significant expression whereas 1 represents significant expression (either over- or under-expression). It was found in [24] that the WNT5A gene is a major driver of processes that lead to metastatic melanoma. We derive the logic relationships and wiring of a 7-gene WNT5A network consisting of genes selected using data analysis and prior biological knowledge: WNT5A, pirin, S100P, RET1, MART1, HADHB, and STC2; for more details about the selection of these genes, see [25, 26].

We assume a model where the target binary gene expression *Y*∈{0,1} is regulated by a binary predictor gene expression vector **X**=(*X*_1_,…,*X*_*d*_)∈{0,1}^*d*^ through the relationship

(31) $$ Y = f(\mathbf{X})\:\oplus\: N,   $$

where *f*:{0,1}^*d*^→{0,1} is a Boolean function, the symbol “ ⊕” indicates modulo-2 addition, and *N*∈{0,1} is a noise Bernoulli random variable, independent from **X**, such that *P*(*N*=0)=*p*. The modulo-2 addition behaves as a XOR operation, which flips the state of the target *Y* when *N*=1, and leaves it unaltered when *N*=0. Hence, *p* quantifies the predictive power of the model: if *p*=1, the system is noiseless and prediction is deterministic, while if *p*<1, there is a degree of indeterminacy in the state of the target given the state of the predictors. This model is studied in detail in [15], where an inference procedure, based on a maximum-likelihood CoD estimator, is proposed to select the unknown Boolean function *f*, assuming that *f* is a member of a candidate model set *F* containing Boolean functions that depend on the same number *k* of essential variables. Each *f* in *F* is specified by (1) a Boolean function *g*:{0,1}^*k*^→{0,1} and (2) the indices for the predicting variable set {*i*_1_,…,*i*_*k*_}⊂{1,…,*d*}, or *wiring*, such that $f(\mathbf {X}) \,=\, g\left (X_{i_{1}},\ldots,X_{i_{k}}\right)$. If the candidate boolean functions *g* belong to a model set *G*, then the total number of possible models is $|G| \times \binom {d}{l}$.

Here, we modify the network inference in [15] to allow the use of the Bayesian CoD estimators described previously. For a given target *Y* and predictor set **X**, we assume Dirichlet prior distributions as in (5). Instead of adopting a non-informative choice of hyperparameters, we employ an “empirical Bayes” approach, where the hyperparameters are estimated in part from the sample data, as described next.

First, it follows from the model in (31) that the parameters *p*_*i*_=*P*(**X**=**x** ^*i*^∣*Y*=0), *q*_*i*_=*P*(**X**=**x** ^*i*^∣*Y*=1), and *c*=*P*(*Y*=0) are given by:

(32) $${} {\fontsize{8.8pt}{9.6pt}{\begin{aligned} p_{i} & \;\propto\; \left(\,p(1-f(\mathbf{x}^{i})) + (1-p) f(\mathbf{x}^{i})\right)P(\mathbf{X} = \mathbf{x}^{i}), \quad i = 1, \ldots, 2^{d}, \\[1ex] q_{i} & \;\propto\;\left(\,p f(\mathbf{x}^{i}) + (1-p)(1-f(\mathbf{x}^{i}))\right)P(\mathbf{X} = \mathbf{x}^{i}), \quad i = 1, \ldots, 2^{d}, \\[1ex] c & \;=\; {\textstyle \sum_{i=1}^{2^{d}}\left(p(1-f(\mathbf{x}^{i}))+ (1-p) f(\mathbf{x}^{i})\right) P(\mathbf{X} =\mathbf{x}^{i})}, \end{aligned}}}   $$

The unknown quantities here are the predictive power *p* and the distribution *P*(**X**) of the predictors. Given the sample data **S**_*n*_={(**X**_1_,*Y*_1_),…,(**X**_*n*_,*Y*_*n*_)}, and a fixed Boolean function *g* and wiring {*i*_1_,…,*i*_*k*_}, *p* can be very effectively estimated by means of the sample frequency [15]:

(33) $$ \hat{p} \,=\, \frac{1}{n}\,\sum_{i=1}^{n} I_{f(\mathbf{X}_{i})=Y_{i}},   $$

The distribution *P*(**X**) could in principle be also estimated from the data using sample frequencies; however, such an estimator can become very poor under small sample sizes and large dimensionality *d*. Therefore, we simply assume a flat distribution *P*(**X**=**x** ^*i*^)=1/2^*d*^, for *i*=1,…,2^*d*^. Substituting this and (33) into (32) gives the values of the hyperparameters used in our experiment:

(34) $$ \begin{aligned} \hat{p}_{i} &\,\propto\, \left(\,\hat{p}(1-f(\mathbf{x}^{i})) + (1-\hat{p}) f(\mathbf{x}^{i})\right), \quad i = 1, \ldots, 2^{d} \\[1ex] \hat{q}_{i} &\,\propto\,\left(\,\hat{p} f(\mathbf{x}^{i}) + (1-\hat{p})(1-f(\mathbf{x}^{i}))\right), \quad i = 1, \ldots, 2^{d} \\[1ex] \hat{c} &\,=\, {\textstyle (1/2^{d})\,\sum_{i=1}^{2^{d}}\left(\hat{p}(1-f(\mathbf{x}^{i}))+ (1-\hat{p}) f(\mathbf{x}^{i})\right)}, \end{aligned}   $$

Recall from Section 3 that the shape of the Dirichlet prior distribution is determined by the hyperparameters through a location parameter, called the base measure, and a concentration parameter. Our strategy is to set up the estimates in (34) as the base measure, so that the Dirichlet priors are concentrated around them, to a degree specified by the concentration parameter. Formally, the hyperparameters are set to: $\{\alpha _{1}, \dots, \alpha _{2^{d}}\} = \{ \lceil \hat {p}_{1} \Delta \rceil, \ldots, \lceil \hat {p}_{2^{d}} \Delta \rceil \}$, $\{\beta _{1}, \dots, \beta _{2^{d}}\} = \{ \lceil \hat {q}_{1} \Delta \rceil, \ldots, \lceil \hat {q}_{2^{d}} \Delta \rceil \}$, $\alpha = \lceil \hat {c} \Delta \rceil $ and $\beta = \lceil (1-\hat {c}) \Delta \rceil $, where ⌈*x*⌉ gives the smallest integer larger or equal to *x*. The value of *δ* is tuned by the experimenter, either manually or using a data-driven procedure.

We are now ready to state the procedure to select a function *f* in *F*, consisting of a *k*-predictor Boolean function *g* and its wiring.

### Bayesian model selection procedure

1. For each of the Boolean functions *g*∈*G*, compute the prior hyperparameters as described earlier. Obtain the MMSE Bayesian CoD / OBP CoD estimate under each of the $\binom {d}{k}$ possible wirings. Pick the wiring for *g* that produces the largest CoD estimate. Ties, if any, are broken randomly.
2. Among the |*G*| pairs of Boolean function *g* and wiring obtained in the previous step, select the one that produces the largest predictive power estimate $\hat {p}$. Ties, if any, are broken randomly.

In our experiment with the 7-gene WNT5A network, we consider in turn each gene as a target and the remaining six genes as predictors (so that a gene cannot be a predictor of itself). Hence, *d*=6. In addition, we assume that that each gene is predicted by three genes out of the six predictors. Therefore, *k*=3 and there are $\binom {6}{3}=20$ possible wirings for each target gene. The set *G* contains all 218 Boolean functions of exactly three essential variables (this is less than the full set of $2^{2^{3}} = 256$ 3-input Boolean functions since those that are reducible to 0-, 1-, and 2-input logics are not considered). We set *Δ*=1.0 and apply the proposed Bayesian model selection procedure to infer a gene regulatory network for the MMSE and OBP CoD estimators. We also obtain the gene regulatory network produced by employing the standard model selection procedure, which picks the predictor set (among all $\binom {6}{3} = 20$ choices, in this case) with the largest estimated resubstitution CoD [25].

**Fig. 6:**
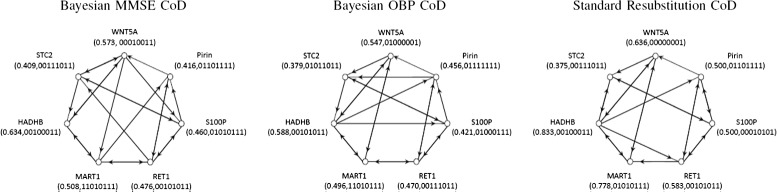
Gene regulatory networks inferred using the Bayesian model selection procedure for the Bayesian MMSE CoD and OBP CoD and the standard model selection procedure using the resubstitution CoD

**Fig. 7:**
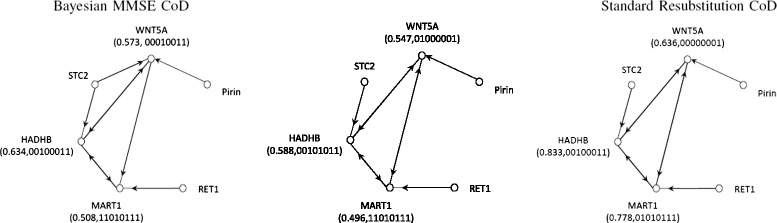
Gene regulatory networks inferred using the Bayesian model selection procedure for the Bayesian MMSE CoD and OBP CoD and the standard model selection procedure using the resubstitution CoD, corresponding to the top three predicted relationships according to CoD magnitude

The results are presented in Fig. 6. The diagrams represent the predicted logic functions as binary strings (in the usual logic table order; e.g., AND = 00000001) and the predicted wirings as oriented edges, and, in addition, the estimated CoD in each case is displayed. We can see that the predicted logic functions and wirings for the three networks are similar, especially in the cases of the OBP and resubstitution CoD networks. If one considers only the three top predicted relationships according to CoD magnitude, one obtains the diagrams depicted in Fig. 7, which show that the same network is inferred by the OBP and resubstitution CoDs, which differ from the network obtained with the MMSE CoD by only a single arrow shift in the wirings (the inferred logics in all three cases are also very similar, differing by only a few bit shifts). The important difference between the Bayesian and standard approaches that can be observed from this experiment is in the estimated CoD magnitudes: those estimated with the standard resubstitution CoD tend to be much larger than the ones estimated with the Bayesian CoDs. This reflects the optimistic bias that tends to be displayed by resubstitution [27], a problem that is avoided by the Bayesian CoD estimators.

## Conclusions

We introduced a Bayesian framework for the estimation of the CoD in a discrete prediction setting and analyzed the accuracy of the proposed Bayesian MMSE and OBP CoD estimators based on fixed and random parameters, using analytical and simulation methods. We also compared the accuracy of the two Bayesian CoD estimators against those of several classical CoD estimators, based on resubstitution, leave-one-out, bootstrap, and cross-validation prediction error estimation. Our results indicated that the Bayesian MMSE CoD estimator has the best performance with zero bias and least RMS, when averaged over all distributions and sample data, whereas, for fixed distributions, we conclude that priors with higher densities around the fixed distributions present better accuracy with less RMS. It is also interesting to see that the OBP CoD estimator, one with very simple calculation, can beat the Bayesian MMSE CoD estimator when using low-variance priors with higher densities around the parameters of the fixed distributions. Furthermore, we proposed an approach for inference of gene regulatory networks based on the proposed Bayesian CoD estimators, and applied it to the inference of a 7-gene regulatory network using melanoma data. We observed that the inferred boolean functions and wirings were similar for both CoD Bayesian estimators. Interestingly, the network inferred with the OBP CoD estimator was very close to the network obtained with the standard inference method based on the resubstitution CoD estimator; however, the magnitude of the CoDs were larger in the latter case, which is consistent with the fact that resubstitution tends to be optimistic. We hope that this paper will provide a theoretical foundation for further work on Bayesian estimation methodologies for the inference of gene regulatory networks. The issue of obtaining informative priors based on established biological knowledge about regulatory relationships, which was not addressed in detail here, is one that deserves careful consideration in future work on this topic.

## Endnote

^1^ A proof of this fact is given in the Appendix of [14].

## Appendix A: the beta distribution

If *X*∼Beta(*a,b*), where *a,b*>0, then the probability density function of *X* is given by

(35) $$ f_{X}(u)\,=\, \frac{1}{\mathrm{B}(a,b)} u^{a-1}(1-u)^{b-1},\quad 0<u<1,  $$

where the normalizing term B(*a,b*) is known as the Beta function:

(36) $$ \mathrm{B}(a,b) \,=\, {\int_{0}^{1}} u^{a-1}(1-u)^{b-1} \,du,  $$

Clearly, *B*(*a,b*)=*B*(*b,a*).

For *k*>−*a* and *l*>−*b*,

(37) $$\begin{array}{@{}rcl@{}} E\left[X^{k}(1-X)^{l}\right] \,&=&\, {\int_{0}^{1}} u^{k}(1-u)^{l} f(u)\,du \notag\\ \,&=&\, \frac{1}{\mathrm{B}(a,b)}{\int_{0}^{1}} u^{a+k-1}(1-u)^{b+l-1} \,du \notag\\ \,&=&\, \frac{\mathrm{B}(a+k,b+l)}{\mathrm{B}(a,b)}, \end{array} $$

For example, *E*[ *X*]=*B*(*a*+1,*b*)/*B*(*a,b*)=*a*/(*a*+*b*) (the second equality can be proved using the definition of the Beta function in terms of the Gamma function and the properties of the latter [28]). Similarly, *E*[ 1/*X*]=*B*(*a,b*−1)/*B*(*a,b*)=(*a*+*b*−1)/(*b*−1), provided that *b*>1.

The *incomplete* Beta function is defined as

(38) $$ \text{IB}(x;a,b)\,=\, {\int_{0}^{x}} u^{a-1}(1-u)^{b-1} \,du,\quad 0 \leq x \leq 1,  $$

Notice that B(*a,b*)=IB(1;*a,b*).

It is easy to verify that

(39) $${} {\fontsize{8.8pt}{9.6pt}{\begin{aligned} P(X \leq x) \,=\, \frac{\text{IB}(x;a,b)}{\mathrm{B}(a,b)} \quad \text{and}\quad P(X > x) \,=\, \frac{\text{IB}(1-x;b,a)}{\mathrm{B}(a,b)}, \end{aligned}}}  $$

Finally, for *k*>−*a* and *l*>−*b*,

(40) $$\begin{array}{@{}rcl@{}} E\left[X^{k}(1-X)^{l}I_{X\leq x}\right] \,&=&\, \frac{\text{IB}(x; a+k,b+l)}{\mathrm{B} (a,b)}I_{x<1} \,\notag\\&&+\, \frac{\mathrm{B}(a+k,b+l)}{\mathrm{B}(a,b)}I_{x \geq 1}, \end{array} $$

which follows easily from the definitions of the Beta density and the incomplete Beta function, and the fact that *X*∈[ 0,1]. In particular, if *x*≥1, then *E*[*X* ^*k*^(1−*X*)^*l*^ *I*_*X*≤*x*_]=*E*[*X* ^*k*^(1−*X*)^*l*^].

Clearly, all the previous quantities can be computed in terms of the incomplete beta function, an expression of which is given by the next result.

### **Theorem1**.

If *X*∼Beta(*a,b*), then

(41) $$ \text{IB}(x;a,b)\,=\, \sum_{i=0}^{P} r_{i}(a,b)\, x^{a+i},   $$

where *P*=*b*−1 and

(42) $$ r_{i}(a,b) \,=\, \frac{(-1)^{i}}{a+i}\binom{b-1}{i}, \quad i =0,\ldots,b-1,  $$

if *b* is an integer, or *P*=*∞* and

(43) $${} {\fontsize{8.8pt}{9.6pt}{\begin{aligned} r_{i}(a,b) \,=\, \frac{(-1)^{i}}{a+i} \frac{(b-1)(b-2)\cdots(b-i+1)}{i!}, \quad i =1,2,\ldots, \end{aligned}}}  $$

otherwise.

### *Proof*.

When *b* is a positive non-integer (that is, ⌊*b*⌋>0), we have, by using the Taylor series expansion,

(44) $$ (1-x)^{b-1} \,=\, \sum_{i=0}^{\infty}(-1)^{i} \binom{b-1}{i} x^{i}.  $$

Note that ⌊*b*⌋ denotes the largest integer that is less than b. Therefore,

(45) $$ \begin{aligned} \text{IB}(k;a,b)& = {\int_{0}^{k}} \sum_{i=0}^{\infty}(-1)^{i} \binom{b-1}{i} x^{a+i-1} dx. \end{aligned}   $$

To interchange the integration and summation in (45), we need to construct a sequence of measurable functions *g*_*i*_(*x*), *i*=0,1,…,*∞*, that satisfy the following three conditions:

- (i) $\left |(-1)^{i} \binom {b-1}{i} x^{a+i-1} \right | \leq g_{i}(x)$, for all *k* and almost all *x*;
- (ii) $\sum _{i=0}^{\infty } g_{i}(x)$ converges for almost all *x*;
- (iii) $\sum _{i=0}^{\infty } {\int _{0}^{1}} g_{i}(x) dx < \infty $.

Let $ g_{i}(x) = \left | \binom {b-1}{i} \right | x^{a+i-1}, i = 0, \ldots, \infty $, and obviously the condition (i) is satisfied.

For 0≤*x*<1,

(46) $$ \sum_{i=0}^{\infty} g_{i}(x) = \sum_{i=0}^{\lfloor b \rfloor} g_{i}(x) + \sum_{i=\lfloor b \rfloor + 1}^{\infty}g_{i}(x),  $$

where

(47) $${} {\fontsize{8.8pt}{9.6pt}{\begin{aligned} \sum_{i=\lfloor b \rfloor + 1}^{\infty}g_{i}(x) & = \sum_{i=\lfloor b \rfloor + 1}^{\infty} \left \{ \prod_{j=1}^{\lfloor b \rfloor} \left|\frac{b}{j}-1\right| \times \prod_{j=\lfloor b \rfloor+1 }^{i} \left|\frac{b}{j}-1\right| \times x^{a+i-1} \right \} \\ &= \sum_{i=\lfloor b \rfloor + 1}^{\infty} \left \{ \prod_{j=1}^{\lfloor b \rfloor} \frac{b-j}{\lfloor b \rfloor-j+1} \times \prod_{j=\lfloor b \rfloor+1 }^{i} \left(1-\frac{b}{j}\right)\right.\\&\qquad\qquad \times\left. x^{a+i-1} \vphantom{\left \{ \prod_{j=1}^{\lfloor b \rfloor} \frac{b-j}{\lfloor b \rfloor-j+1} \times \prod_{j=\lfloor b \rfloor+1 }^{i} \left(1-\frac{b}{j}\right)\right.}\right\} \\ & < \sum_{i=\lfloor b \rfloor + 1}^{\infty}x^{a+i-1} \,\, (\text{Since} \,\, \lfloor b \rfloor + 1> b)\\ & = \frac{x^{a+\lfloor b \rfloor}}{1-x} < \infty, \end{aligned}}}  $$

and thus the condition (ii) is satisfied.

(48) $$ \sum_{i=0}^{\infty} {\int_{0}^{1}} g_{i}(x) dx = \frac{1}{a} + \sum_{i=1}^{\infty} \prod_{j=1}^{i} \left|\frac{b}{j}-1\right|\frac{1}{a+i},  $$

Let $ \prod _{j=1}^{i} \left |\frac {b}{j}-1\right |\frac {1}{a+i} = z_{i} (i = 1, 2, \ldots, \infty)$. Since

(49) $${} {\lim}_{i \rightarrow \infty }i \cdot \left(\frac{z_{i}}{z_{i+1}}-1\right) ={\lim}_{i \rightarrow \infty }\frac{(b-i)(a+2i+1)}{(b-i+1)(a+i) }= 2 > 1,  $$

$\sum _{i=0}^{\infty } {\int _{0}^{1}} g_{i}(x) dx$ converges by Raabe’s test [29].

Now we can interchange the integration and summation in (45), and we have

(50) $$ \begin{aligned} \text{IB}(k;a,b)& =\sum_{i=0}^{\infty} (-1)^{i} \binom{b-1}{i} {\int_{0}^{k}}x^{a+i-1} dx \\ &=\sum_{i=0}^{\infty} \frac{(-1)^{i} k^{a+i}}{a+i} \binom{b-1}{i}, \end{aligned}   $$

When *b* is an integer, we have $(1-x)^{b-1} \,=\, \sum _{i=0}^{b-1}(-1)^{i} \binom {b-1}{i} x^{i}$, and it is easy to show that

(51) $$ \text{IB}(k;a,b) =\sum_{i=0}^{b-1} \frac{(-1)^{i} k^{a+i}}{a+i} \binom{b-1}{i},  $$

Notice that $B(a,b) = \sum _{i=0}^{P} r_{i}(a,b)$. Note also that the general case reduces to the special case if *b* is an integer. An equivalent expression can be derived where *a* appears in the binomial coefficient instead, which can then be used if *a* is an integer. If neither *a* nor *b* are integers, an approximation can be obtained by truncating the resulting infinite series, or by using a numerical software package.

If both *a* and *b* are integers, then IB(*x*;*a,b*) reduces to a polynomial in *x*. Otherwise, it is a simple matter to replace the finite summations by infinite series as specified in Theorem 1.

## Appendix B: the Dirichlet distribution

Consider a random vector **X**=(*X*_1_,…,*X*_*K*_), with *K*≥2, defined over the (*K*−1)-simplex

$$\begin{aligned} S_{K-1} &= \left\{(X_{1},\ldots,X_{K}) \in R^{K} \mid X_{i}\geq 0,\: i=1,\ldots,K,\:X_{1}\right.\\&\quad +\left.\cdots+X_{K} = 1\vphantom{\left\{(X_{1},\ldots,X_{K}) \in R^{K} \mid X_{i}\geq 0,\: i=1,\ldots,K,\:X_{1}\right.}\right\}, \end{aligned} $$

If **X**∼Dirichlet(*α*_1_,…,*α*_*K*_), where *a*_*i*_>0, for *i*=1,…,*K*, then the probability density function of **X** is given by

(52) $${} {\fontsize{8.4pt}{9.6pt}{\begin{aligned} {f_{\mathbf{X}}(x_{1},\ldots,x_{K})\,=\, \frac{1}{\mathrm{B}(a_{1},\ldots,a_{K})}\: \prod_{i=1}^{K} x_{i}^{a_{i}-1}},\quad (x_{1},\ldots,x_{K}) \in S_{K-1}, \end{aligned}}}  $$

where the normalizing term B(*a*_1_,…,*a*_*K*_) is the multivariate generalization of the Beta function:

(53) $$ \mathrm{B}(a_{1},\ldots,a_{K}) \,=\, \int_{S_{K-1}} \prod_{i=1}^{K} x_{i}^{a_{i}-1}\, d\mathbf{x},  $$

The shape of the Dirichlet distributions controlled by the *concentration parameter* $\Delta = \sum _{i=1}^{K} a_{i}$ and the *base measure* $\left (a_{1}^{\prime }, \ldots, a_{K}^{\prime }\right) = (a_{1}/\Delta, \ldots, a_{K}/\Delta)$. Note that the base measure is a valid discrete probability measure. It can be shown easily that

$$E[\mathbf{\!X}] = (a_{1}^{\prime}, \ldots, a_{K}^{\prime}), $$

so that the base measure provides the “central” value around which **X** is distributed. In particular, large components in the base measure bias the distribution in their direction.

The concentration parameters, on the other hand, control the variance of the distribution around the base measure, with large values indicating smaller variance. In fact, it can be shown that [30]

(54) $$ \begin{aligned} \text{Var}(X_{i}) &\,=\, \frac{a_{i}^{\prime} (1-a_{i}^{\prime})}{\Delta+1} \\ \text{Cov}(X_{i},X_{j}) &\,=\, \frac{-a_{i}^{\prime}\, a_{j}^{\prime}}{\Delta+1}, \quad\text{for} i \neq j, \end{aligned}  $$

From the previous equations, one can see that, as *δ* approaches infinity, variances converge to zero and **X** becomes equal to the base measure with probability 1; in addition, covariances also go to zero, rendering the components of **X** uncorrelated. The special case *a*_*i*_=1, for all *i*=1,…,*K* corresponds to a uniform over *S*_*K*−1_. This corresponds to a uniform base measure and concentration parameter *Δ*=*K*. If the base measure is not uniform but *Δ*=*K*, the distribution is approximately uniform. For *δ* approaching zero, the distribution becomes concentrated at the boundary of the simplex.

Summing up, large *δ* implies large probability density around the base measure, *Δ*=*K* implies a nearly uniform distribution, whereas *δ* close to zero produces sparse sample vectors with most of the components close to zero.

The Dirichlet distribution is the multivariate generalization of the Beta distribution, in the sense that the components of a Dirichlet-distributed vector **X**=(*X*_1_,…,*X*_*K*_) are Beta distributed: *X*_*i*_∼Beta(*a*_*i*_,*Δ*−*a*_*i*_), for *i*=1,…,*K*. Notice that in the case *K*=2 the Dirichlet distribution essentially reduces to the Beta distribution.

## References

[1] Kauffman S (1969). Metabolic stability and epigenesis in randomly constructed genetic nets. J Theor. Biol.

[2] Kauffman S (1993). *The Origins of Order: Self-Organization and Selection in Evolution*.

[3] Bornholdt S (2008). Boolean network models of cellular regulation: prospects and limitations. J. R. Soc. Interface.

[4] Albert R, Othmer H (2003). The topology of the regulatory interactions predicts the expression pattern of the segment polarity genes in drosophila melanogaster. J. Theor. Biol.

[5] Li F, T Long YLu, Ouyang Q, Tang C (2004). The yeast cell-cycle network is robustly designed. Proc. Natl. Acad. Sci. U.S.A..

[6] Faure A, Naldi A, Chaouiya C, Thieffry D (2006). Dynamical analysis of a generic boolean model for the control of the mammalian cell cycle. Bionformatics.

[7] Dougherty ER, Kim S, Chen Y (2000). Coefficient of determination in nonlinear signal processing. EURASIP J. Signal Process.

[8] Kim S, Dougherty ER, Chen Y, Sivakumar K, Meltzer P, Trent JM, Bittner M (2000). Multivariate measurement of gene expression relationships. Genom.

[9] Zhou X, Wang X, Dougherty ER (2003). Binarization of microarray data based on a mixture model. Mol. Cancer Ther.

[10] Kim S, Dougherty ER, Bittner ML, Chen Y, Sivakumar K, Meltzer P, Trent JM (2000). General nonlinear framework for the analysis of gene interaction via multivariate expression arrays. J. Biomed. Opt.

[11] Shmulevich I, Dougherty ER, Kim S, Zhang W (2002). Probabilistic Boolean networks: a rule-based uncertainty model for gene regulatory networks. Bioinforma.

[12] Martins D, Braga-Neto U, Hashimoto R, Bittner M, Dougherty ER (2008). Intrinsically multivariate predictive genes. IEEE J. Sel. Top. Sign. Proces.

[13] Chen T, Braga-Neto UM (2015). Statistical detection of intrinsically multivariate predictive genes. IEEE/ACM Trans. Comput. Biol. Bioinform.

[14] T Chen, UM Braga-Neto, Exact performance of CoD estimators in discrete prediction. EURASIP J. Adv. Signal Process (2010). (Article ID 2010:487893).

[15] Chen T, Braga-Neto UM (2013). Maximum-likelihood estimation of the discrete coefficient of determination in stochastic boolean systems. IEEE Trans. Signal Process.

[16] Chen T, Braga-Neto UM (2013). Statistical detection of Boolean regulatory relationships. IEEE/ACM Trans. Comput. Biol. Bioinform.

[17] Dalton LA, Dougherty ER (2011). Bayesian minimum mean-square error estimation for classification error – Part I: Definition and the Bayesian mmse error estimator for discrete classification. IEEE Trans. Signal Process.

[18] Dalton LA, Dougherty ER (2011). Bayesian minimum mean-square error estimation for classification error – Part II: Linear classification of gaussian models. IEEE Trans. Signal Process.

[19] Chen T, Braga-Neto UM (2013). Optimal Bayesian MMSE estimation of the coefficient of determination for discrete prediction. *In Proceedings of the 2013 IEEE International Workshop on Genomic Signal Processing and Statistics (GENSIPS’2013)*.

[20] Dalton LA, Dougherty ER (2013). Optimal classifiers with minimum expected error within a Bayesian framework – Part I: Discrete and gaussian models. Pattern Recogn.

[21] Dalton LA, Dougherty ER (2013). Optimal classifiers with minimum expected error within a Bayesian framework – Part II: Properties and performance analysis. Pattern Recogn.

[22] Devroye L, Gyorfi L, Lugosi G (1996). *A Probabilistic Theory of Pattern Recognition*.

[23] Casella G, Berger R (2002). *Statistical Inference*.

[24] Bittner M, Meltzer P, Chen Y, Jiang Y, Seftor E, Hendrix M, Radmacher M, Simon R, Yakhini Z, Ben-Dor A, Sampas N, Dougherty ER, Marincola F, Wang E, Gooden C, Lueders J, Glatfelter A, Pollock P, Carpten J, Gillanders E, Leja D, Dietrich K, Beaudry C, Berens M, Alberts D, Sondak V, Hayward N, Trent J (2000). Molecular classification of cutaneous malignant melanoma by gene expression profiling. Nature.

[25] Kim S, Dougherty ER, Cao N, Chen Y, Bittner M, Suh E (2002). Can markov chain models mimic biological regulation?. J. Biol. Syst.

[26] Datta A, Choudhary A, Bittner M, Dougherty ER (2003). External control in markovian genetic regulatory networks. Mach. Learn.

[27] Braga-Neto UM, Dougherty ER (2015). *Error Estimation for Pattern Recognition*.

[28] Ross S (1994). *A first course in probability*.

[29] Arfken G (1985). *Mathematical Methods for Physicists*.

[30] Balakrishnan N, Nevzorov V (2003). *A Primer on Statistical Distributions*.

